# Supplementary material for: Ensuring Equitable COVID-19 Vaccine Allocation in New Hampshire: The First Eight Months toward a New Era
Source: Vaccines (Basel). 2022 Aug 29;10(9):1421. doi: 10.3390/vaccines10091421 (PMC9501825; doi:10.3390/vaccines10091421)
Supplement: Supplementary file 1 [file vaccines-10-01421-s001.zip › vaccines-1843494-supplementary/S1.pdf]

NEW HAMPSHIRE
CORONAVIRUS DISEASE 2019
VACCINATION PLAN

March 15, 2021

New Hampshire Department of Health and Human Services

TABLE OF CONTENTS

RECORD OF CHANGES 2
ABBREVIATIONS USED IN THIS DOCUMENT 3
ABOUT THIS DOCUMENT 4
EXECUTIVE SUMMARY 5
SECTION 1: COVID-19 VACCINATION PREPAREDNESS PLANNING 7
SECTION 2: COVID-19 ORGANIZATIONAL STRUCTURE AND PARTNER INVOLVEMENT 8
SECTION 3: PHASED APPROACH TO COVID-19 VACCINATION 11
SECTION 4: CRITICAL POPULATIONS 16
SECTION 5: COVID-19 PROVIDER RECRUITMENT AND ENROLLMENT 19
SECTION 6: COVID-19 VACCINE ADMINISTRATION CAPACITY 22
SECTION 7: COVID-19 VACCINE ALLOCATION, ORDERING, DISTRIBUTION, AND INVENTORY MANAGEMENT 23
SECTION 9: VACCINE ADMINISTRATION DOCUMENTATION AND REPORTING 26
SECTION 10: COVID-19 VACCINATION SECOND-DOSE REMINDERS 28
SECTION 11: COVID-19 REQUIREMENTS FOR IISs OR OTHER EXTERNAL SYSTEMS 29
SECTION 12: COVID-19 VACCINATION PROGRAM COMMUNICATION 30
SECTION 13: REGULATORY CONSIDERATIONS FOR COVID-19 VACCINATION 32
SECTION 14: COVID-19 VACCINE SAFETY MONITORING 34
SECTION 15: COVID-19 VACCINATION PROGRAM MONITORING 35
APPENDIX 1: INCIDENT MANAGEMENT TEAM STRUCTURE 37
APPENDIX 2: VACCINE OPERATIONS SECTION 38
APPENDIX 3: IMMUNIZATION PROGRAM SECTION 40
APPENDIX 4: STATE DISASTER MEDICAL ADVISORY COMMITTEE (SDMAC) 41

RECORD OF CHANGES

Date of original version: 10/16/2020

Date Reviewed	Change Number	Date of Change	Description of Change	Name of Author
10/30/2020	1	10/30/2020	Added Executive Summary	C. Haggerty, E. Daly
12/15/2020<br>01/30/2021<br>03/01/2021	2	03/02/2021	• Added equity considerations under critical populations<br>• Added Workforce Branch in Vaccine Operations Section organizational structure<br>• Updated Phase 1a Guidance<br>• Added Phase 1b Guidance<br>• Added Phase 2 Guidance<br>• Added information on v-safe<br>• Addition of state-managed fixed sites information<br>• Made general updates throughout regarding current status of plan, now that it is being implemented	T. Selembo<br>K. Durzy<br>E. Daly

ABBREVIATIONS USED IN THIS DOCUMENT

ACIP	Advisory Committee on Immunization Practices
CDC	U.S. Centers for Disease Control and Prevention
EUA	Emergency Use Authorization
ESU	Emergency Services Unit
FEMA	Federal Emergency Management Agency
FDA	U.S. Food and Drug Administration
HAN	Health Alert Network
HCP	Healthcare Personnel
IMT	Incident Management Team
LTCF	Long-term Care Facility
MRC	Medical Reserve Corp
NH DHHS	New Hampshire Department of Health and Human Services
NHIIS	New Hampshire Immunization Information System
NHIP	New Hampshire Immunization Program
NH DPHS	New Hampshire Division of Public Health Services
PPE	Personal protective equipment
RPHNs	Regional Public Health Networks
SDMAC	State Disaster Medical Advisory Committee
VAERS	Vaccine Adverse Event Reporting System
VINI	Vaccine and Immunization Network Interface
VTrckS	Vaccine Tracking System

ABOUT THIS DOCUMENT

This plan was developed by the New Hampshire (NH) Department of Health and Human Services (DHHS), Division of Public Health Services (DPHS), Bureau of Infectious Disease Control, Immunization Program. The planning process to develop this plan included a variety of internal and external stakeholders. Content included in this plan was adapted, in part, from the Centers for Disease Control and Prevention's *Vaccination Program Interim Playbook for Jurisdiction Operations*, prior pandemic and outbreak event experience in New Hampshire, preparedness plans, and other resources. The format and content of this plan is in accordance with the specific elements required to be addressed by the Centers for Disease Control and Prevention. The COVID-19 vaccine planning process has required flexibility as guidance and planning assumptions continue to evolve. This plan is intended to represent current plans and strategies as of the date of the document; however, DPHS will adapt its approach as appropriate based on new science or national best practices and guidelines throughout the vaccination initiative.

For questions about this plan, please contact:

For questions about this plan, please contact:
NH Department of Health and Human Services
Bureau of Infectious Disease Control
NH Immunization Program
29 Hazen Drive, Concord, NH 03301
Phone: (603) 271-4482
Email: immunization@dhhs.nh.gov

EXECUTIVE SUMMARY

The Coronavirus Disease 2019 (COVID-19) pandemic has caused substantial morbidity and mortality, and significant economic and social disruption. Through current mitigation efforts, and availability of safe and effective COVID-19 vaccines, New Hampshire's (NH) goal is to decrease disease burden and ensure NH citizens remain healthy and free from disease in every stage of life. This plan was developed by the NH Department of Health and Human Services (DHHS), Immunization Program (NHIP) and informed by the Centers for Disease Control and Prevention's *Vaccination Program Interim Playbook for Jurisdiction Operations*, prior pandemic and outbreak experience in NH, preparedness plans, and the National Academies for Science, Engineering, and Medicine's *A Framework for Equitable Allocation of Vaccine for the Novel Coronavirus.* This plan represents current plans and strategies. NH's approach will be adapted as appropriate based on new science or national best practices and guidelines throughout the vaccination initiative. Key components of NH's plan are summarized here.

SECTION 3: PHASED APPROACH TO COVID-19 VACCINATION: NH's vaccination program is structured around the concept of a phased response, whereby vaccine may initially be limited. A Vaccine Allocation Strategy Branch informs strategies related to equitable dose distribution. Ensuring equitable access to COVID-19 is central to NH's vaccine planning efforts. NH's initial plan included vaccination of the following groups under Phase 1a: High-risk workers in health care facilities, first responders, and older adults in residential care settings. Later phases are outlined in this plan.

SECTION 4: CRITICAL POPULATIONS: Some populations have been disproportionately impacted by COVID-19 and are at increased risk for infection, severe illness, and death. Data has been gathered from multiple sources to determine accurate numbers for distribution planning. Key partnerships have been developed with organizations that serve vulnerable populations to ensure availability of vaccine in these critical populations The Vaccine Operations Section's Communication Branch has developed a comprehensive communication plan that clearly delineates methods for communication with various entities, including the general public and partners that serve critical populations as well as community leaders.

SECTION 5: COVID-19 PROVIDER RECRUITMENT AND ENROLLMENT: NH is a universal childhood vaccine purchase state, with many providers already enrolled in NHIP. A Government/Non-government partnership of distribution has been implemented. Providers identified as vaccinators in the first phase were prioritized for enrollment. Recruitment is being based on utilization in the next phases, as well as creating depth of vaccine providers and decreasing barriers towards immunization. Additionally, fixed sites on the government side of the response were established to supplement these individual vaccine providers. Pharmacies are also recruited to provide additional vaccination services. Vaccine providers are verified through the established, Vaccine Provider Agreement process; provider enrollment data are submitted via CDC's Vaccine Tracking System (VTrckS) and sent to CDC. Ensuring equitable access to COVID-19 vaccine is central to planning efforts, with oversight by the Vaccine Allocation Strategy Branch, and solicitation of feedback from the State Disaster Medical Advisory Committee and the DHHS Office of Health Equity. Key documents leveraged for this work include NH's COVID-19 Equity Response Team report, CDC's COVID-19 Response Health Equity Strategy, the National Academies for Science, Engineering, and

Medicine's *A Framework for Equitable Allocation of Vaccine for the Novel Coronavirus*, and the CDC's Advisory Committee on Immunization Practices' recommendations. Pharmacy distribution plans are reviewed by the Vaccine Operations Section to ensure equitable and fair distribution.

SECTION 8: COVID-19 VACCINE STORAGE AND HANDLING: Prior to the receipt of COVID-19 vaccine, new vaccine providers are required to complete vaccine storage and handling training. The training is conducted via an independent, on-line education portal (CDC TRAIN), webinars, an established Help Line that will include clinical and vaccine management subject-matter experts, and various job aids. COVID-19 vaccine storage and handling capabilities will be evaluated via the COVID-19 specific, CDC Vaccine Provider Agreement and Profile.

SECTION 10: COVID-19 VACCINATION SECOND-DOSE REMINDERS: Individuals are notified when they are eligible for their second dose of COVID-19 vaccine via the NH Immunization Information System's reminder/recall feature, through CDC "shot cards" provided upon initial vaccine receipt, through their medical home's reminder/recall process, or text messaging. State-wide documentation is leveraged to ensure the second dose of a vaccine presentation is the same as the first, observing recommended intervals between vaccine doses.

SECTION 12: COVID-19 VACCINATION PROGRAM COMMUNICATION: NH's COVID-19 vaccination communication plan is continuously updated to include addressing communications with key audiences, identification of effective communication channels, and partner activation for each phase of the COVID-19 Vaccination Program. NH DHHS has established processes for providing crisis and emergency risk communications in an expedited manner. This includes leveraging NH's Joint Information Center and the Health Alert Network (HAN). A member of the Department's Public Information Office leads the vaccine planning Communication Branch.

SECTION 14: COVID-19 VACCINE SAFETY MONITORING: Through the vaccine provider enrollment process, NH ensures enrolled vaccination providers understand the requirements and process for reporting adverse events to the Vaccine Adverse Event Reporting System. Information is also made available through NHIP's website, social media platforms, and quarterly NHIP conference calls.

SECTION 15: COVID-19 VACCINATION PROGRAM MONITORING: NH has implemented methods and procedures for monitoring progress in the COVID-19 Vaccination Program implementation. Vaccine ordering and distribution is approved at the state level and vaccine wastage is monitored through standard, established procedures. Methods and procedures for monitoring resources are in place, including monitoring the budget, staffing, and supplies. Reception of public communication messages is monitored through the public information office, media inquiries, and social media. Program metrics monitored include vaccination provider enrollment, doses distributed, doses administered, and vaccination coverage. Bi-directional communication pathways are established and leveraged to provide real-time feedback to inform NH's COVID-19 vaccine response efforts.

SECTION 1: COVID-19 VACCINATION PREPAREDNESS PLANNING

A. Vaccine Planning Process

Public health and healthcare organizations in New Hampshire have been planning for pandemic vaccine distribution for nearly two decades. There are a number of plans in place to support vaccination during a pandemic. We also have the experience of distributing vaccine during the H1N1 pandemic and other public health emergencies. NH DHHS has leveraged these plans, our existing strong partnerships, and our prior experience in order to mount a comprehensive vaccination program in response to COVID-19. For COVID-19 specifically, NH DHHS has been planning since June 2020 for the eventual availability of a safe and effective COVID-19 vaccine. This planning has been accomplished through formation of a structured planning team with feedback from partners.

B. Continuous Quality Improvement

Maintaining a flexible approach and willingness to adapt vaccination initiative strategies throughout the response is important to problem solving and ensuring an effective program. Continuous quality improvement is conducted through established strategic planning, participation in roundtables, preparedness exercises, including annual school-based flu vaccine clinics and after-action meetings, and regular engagement with internal and external partners. This approach will continue throughout the COVID-19 response efforts, following traditional incident command structure. The following historical events have also contributed to the overall planning process:

- Key findings from the 2009 Influenza A H1N1 pandemic vaccination initiative
- Key findings from the 2019 Crimson Contagion high-threat infectious disease national exercise
- Federal Vaccine Tabletop Exercise, 09/17/2020 (Partners: FEMA, NH DHHS/DPHS, ESU)
- Annual School-Based Flu Clinics held annually for the last ten years via Regional Public Health Networks (RPHNs)
- Immunization Program weekly planning sessions, commencing July 7, 2020

SECTION 2: COVID-19 ORGANIZATIONAL STRUCTURE AND PARTNER INVOLVEMENT

A. Organizational Structure

New Hampshire's COVID-19 Vaccination Plan requires cooperation between government and non-government entities in order to ensure a successful distribution methodology. This includes hospital organizations, medical homes (primary care providers, prisons, Veteran's Association, home health, community health centers, etc.), emergency medical services, long-term care and assisted living facilities, and organizations supporting individuals experiencing housing insecurity. It is expected that New Hampshire's distribution will include vaccinating approximately 75% of the population through external stakeholders and 25% via government response. Communication pathways are established through a formalized communication branch to delineate messages between general public and external vaccinators and government vaccinators.

B. Internal COVID-19 Vaccination Planning Structure

The organizational structure follows the DPHS' Incident Management Team (IMT) (Appendix 1), with the Vaccine Operations Section coming under the Incident Commander (Appendix 2). The Vaccine Operations Section includes seven branches:

1) Vaccine Allocation Strategy
2) Communication
3) Logistics
4) Workforce
5) Vaccine Accountability
6) Vaccine Documentation
7) Medical Direction

C. Committee of Key Internal Leaders and External Partners

Initial COVID-19 vaccine planning included the Immunization Program team (Appendix 3); and expanded to include representation from:

- CDC Public Health Associate Program
- Chief Medical Officer
- Chief, Bureau of Housing Support
- Chief, Bureau of Infectious Disease Control
- NH Homeland Security and Emergency Management
- NH Hospital Association
- NH Medical Society

- Contracted Epidemiologists
- Chief, Fire Standards & Training/ Emergency Medical Services
- Deputy Public Health Director
- DHHS Emergency Services Unit
- DPHS Incident Management Team
- Governor's COVID Equity Response Team
- Granite State Health Care Coalition
- NH Health Care Coalition
- NH National Guard
- NH State Epidemiologist
- NH Deputy State Epidemiologist
- Office of Medicaid
- Office of Professional Licensure
- Public Health Director / State Health Official
- Public Health Emergency Preparedness Director
- Public Health Information Office
- Regional Public Health Network Coordinators
- State Disaster Medical Advisory Council

D. Additional Relevant Expertise

The NH Crisis Standards of Care Committee was formed to provide input on the equitable distribution of durable medical equipment, medications and other scarce medical resources during the COVID-19 pandemic. This expanded into the development of the State Disaster Medical Advisory Committee (SDMAC) group, and has assumed advisory capacity for COVID-19 vaccine allocation related response efforts (Appendix 4).

E. Local Coordination

New Hampshire has a centralized public health structure with disease control authority resting with the DHHS Commissioner. There are two local health departments in the state's largest cities, Manchester and Nashua. In order to provide local public health response capacity, there are 13 Regional Public Health Networks (RPHNs), that plan, train for, and respond to public health emergencies based on CDC's 15 Preparedness Capabilities. Their role is to improve the overall preparedness and resiliency of communities, while also developing specific emergency response capabilities across the public health, health care, and behavioral health systems, which includes extensive planning and exercising for mass vaccination initiatives. During COVID-19 response efforts, the RPHNs are conducting COVID-19 vaccination clinics within their regions. Their response effort participation is coordinated through the state ICS structure. The RPHNs have plans in place to communicate with key stakeholders and local officials within their regions and with the State of New Hampshire during emergency response and mass vaccination campaigns.

F. Tribal Coordination

New Hampshire does not have state or federally recognized Native American tribes.

G. Key Partners for Critical Populations

Pharmacies, correctional facilities, Federally-Qualified Health Centers (FQHCs) and additional external partners that represent homebound seniors, group homes and minority populations, are being incorporated into New Hampshire's COVID-19 Vaccination Plan through formalized vaccine provider agreements. Once the provider meets the requirements of the agreement and submits it to the NH Immunization Program (NHIP), these providers are created in New Hampshire's Immunization Information System (NHIIS), provided training on how to order and document vaccination as well as creating facilities and end user profiles in NHIIS. These processes will allow for the ordering and documentation of COVID-19 vaccine by pharmacies and correctional facilities. Homeless shelters, assisted living facilities and other populations that have barriers to care, such as those with substance use disorder, are an RPHN focal area. RPHNs will bring vaccine and vaccinators to these locations and have experiencing reaching vulnerable populations from our recent 2019 statewide hepatitis A vaccination campaign.

SECTION 3: PHASED APPROACH TO COVID-19 VACCINATION

A. Vaccination Program Strategy

New Hampshire's vaccination program is structured around the concept of a phased response, whereby vaccine may be available as follows:

Phase 1: Potentially Limited Doses Available

Phase 2: Large Number of Doses Available, Supply Likely to Meet Demand

Phase 3: Likely Sufficient Supply, Slowing Demand

A Vaccine Allocation Strategy Branch was developed to inform strategies related to equitable dose distribution. This branch coordinates with the aforementioned SDMAC group for external perspective and recommendations. The final approval of vaccine distribution is informed by recommendations from the Centers Disease Control and Prevention (CDC), Advisory Committee on Immunization Practices (ACIP), the New Hampshire Vaccine Allocation Strategy and Medical Direction Branches, NH DHHS Commissioner and the Governor's Office. Allocations are distributed utilizing a combination of fixed and mobile government distribution sites, as well as leveraging hospitals, medical home providers, and pharmacies.

Ensuring equitable access to COVID-19 is central to New Hampshire's vaccine planning efforts and decisions are guided by federal guidance with adaptions made based on local conditions and vulnerable populations. Our initial planning efforts were guided by the National Academies for Science, Engineering, and Medicine's *A Framework for Equitable Allocation of Vaccine for the Novel Coronavirus* and are updated as needed based on any additional federal guidance that may be issued. We adopted the *Framework's* guiding ethical principles in New Hampshire, which include:

- Maximum benefit encompasses the obligation to protect and promote the public's health and its socioeconomic well-being in the short and long term.
- Equal concern requires that every person be considered and treated as having equal dignity, worth, and value.
- Mitigation of health inequities includes the obligation to explicitly address the higher burden of COVID-19 experienced by the populations affected most heavily, given their exposure and compounding health inequities.

Additionally, we adopted the *Framework*'s guiding procedural principles, which include:

- Fairness requires engagement with the public, particularly those most affected by the pandemic, and impartial decision making about and evenhanded application of allocation criteria and priority categories.
- Transparency includes the obligation to communicate with the public openly, clearly, accurately, and straightforwardly about the allocation framework as it is being developed, deployed, and modified.
- Evidence-based expresses the requirement to base the allocation framework, including its goal, criteria, and phases, on the best available and constantly updated scientific information and data.

These guiding principles were used to develop New Hampshire's phased allocation strategy outlined in the figure below.

Figure: New Hampshire's Phased Approach to Vaccine Allocation for COVID-19

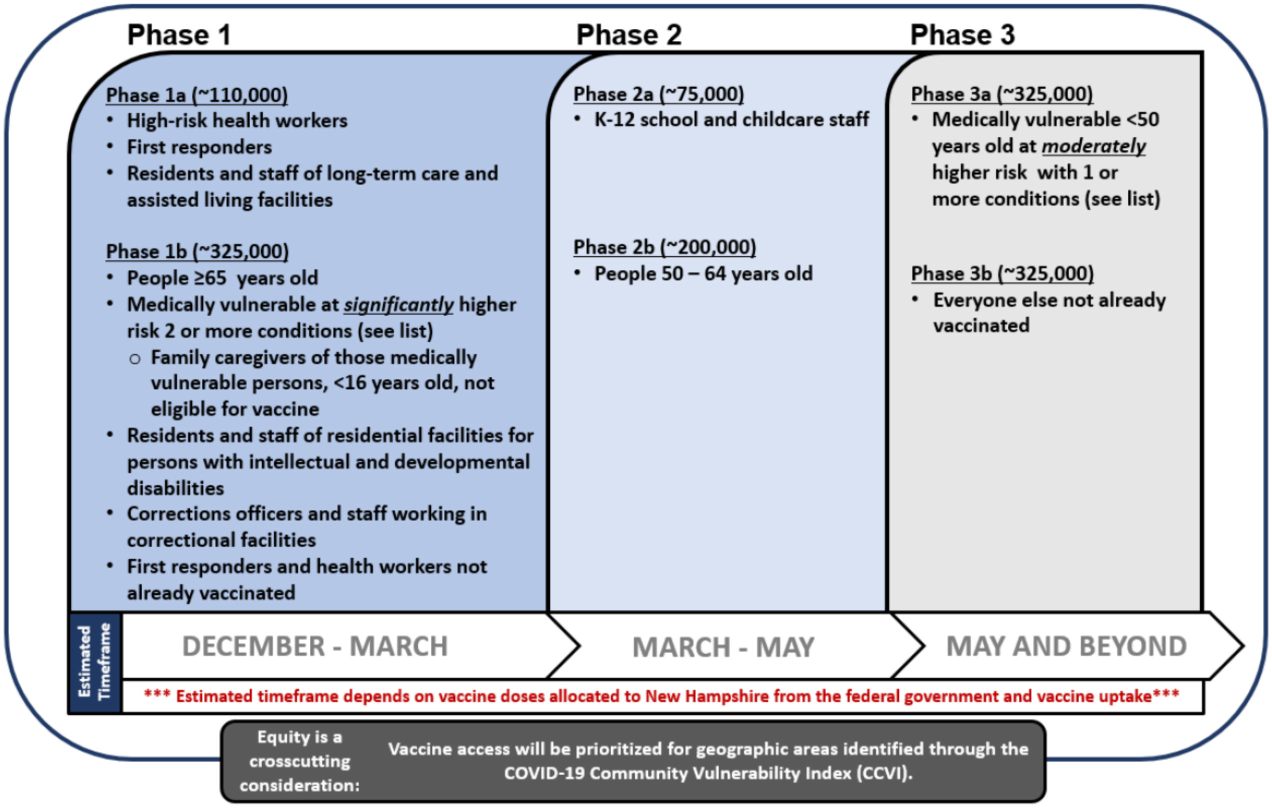

Note: The estimated timeline is also subject to change depending on the number of doses allocated to New Hampshire and the number of people in each group, and how many get vaccinated.

List of Underlying Medical Conditions (modified from CDC):

Phase 1b: Two or more conditions
Phase 3a: One condition

- Cancer
- Chronic Kidney Disease
- COPD (Chronic Obstructive Pulmonary Disease) and other high-risk pulmonary disease
- Down Syndrome
- Heart Conditions, such as heart failure, coronary artery disease, or cardiomyopathies
- Immunocompromised states
- Obesity (body mass index of 30 kg/m or higher)
- Pregnancy
- Sickle cell disease
- Type 2 Diabetes Mellitus

Note: DPHS allows a health care provider to vaccinate any patient assessed to have significant risk for severe illness due to co-morbidities, even if not listed here. This list does not include every condition that might increase one's risk for developing severe illness from COVID-19, such as those for which evidence may be limited (e.g., rare conditions or combinations of conditions).

For Phase 1a, New Hampshire included older adults living in residential care settings (e.g. nursing homes and assisted living facilities). New Hampshire's Phase 1a vaccination allocation strategy is outlined in greater detail in the Phase 1a Allocation Guidelines document.

Figure: New Hampshire's Phase 1a

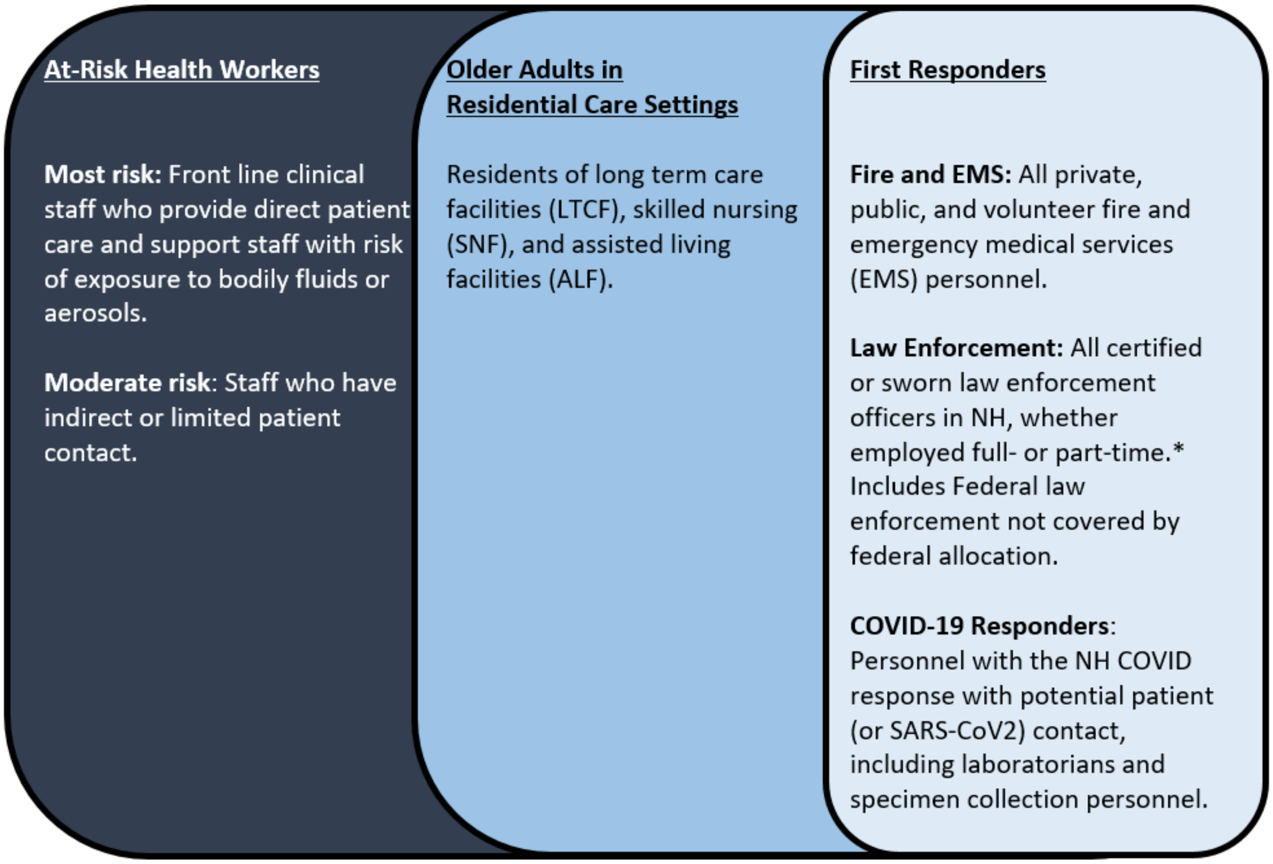

*Does not include officers working in correctional facilities, which are included in a later phase.

The Vaccine Allocation Strategy Branch gathered data from hospital organizations, long-term care and assisted living facilities, first responders (to include Fire, EMS, and Police). Data provided initial estimates for each of the Phase 1a population groups. This coincided with rough estimates for New Hampshire's COVID-19 vaccine distribution over the initial 2months for Phase 1a, which included an estimated 110,000 people.

For Phase 1b, New Hampshire included the groups listed in the figure below. There are approximately 325,000 people estimated in Phase 1b based on U.S. Census Bureau data and the estimated proportion of the population with significant medical vulnerability. New Hampshire's Phase 1b vaccination allocation strategy is outlined in Phase 1b Allocation Guidelines online.

Figure: New Hampshire's Phase 1b

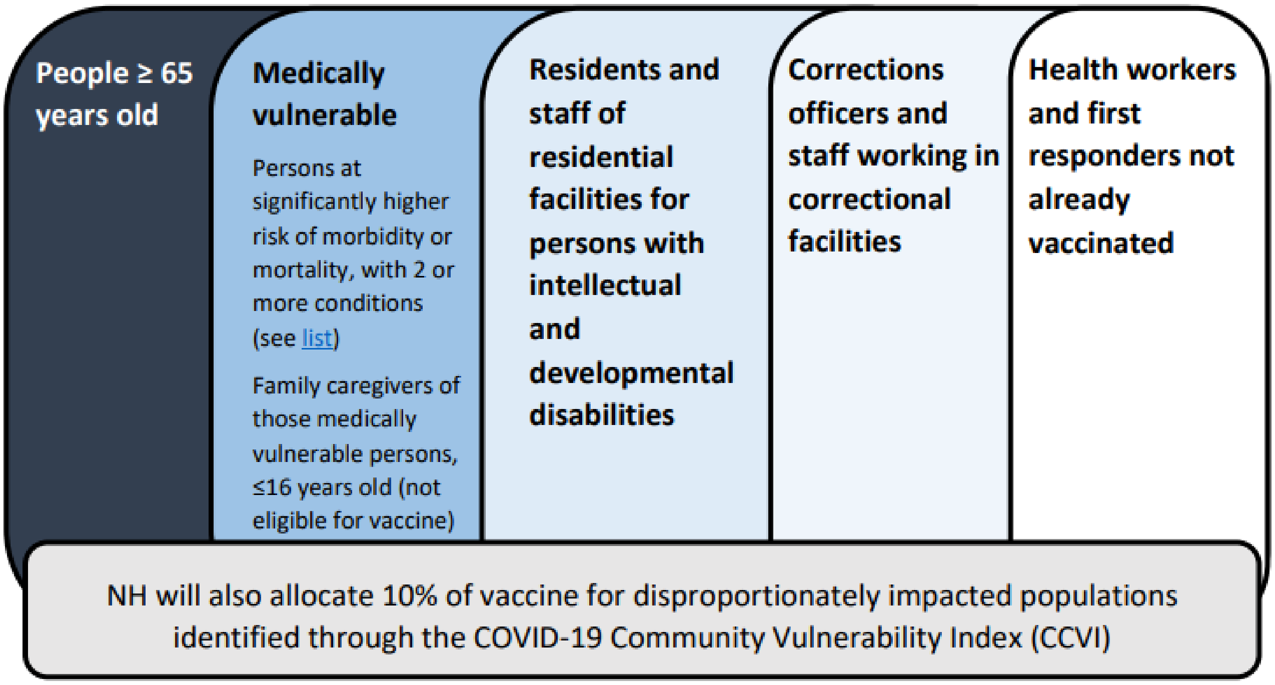

For Phase 2a, New Hampshire included K-12 school staff, childcare providers, and youth camp staff working in New Hampshire. There are approximately 75,000 people estimated to be included in Phase 2a. For Phase 2b, New Hampshire has included persons 50 – 64 years old. There are approximately 200,000 people estimated to be included in Phase 2b. New Hampshire's Phase 2 vaccination allocation strategy is outlined in Phase 2 Allocation Guidelines available online.

Figure: New Hampshire's Phase 2

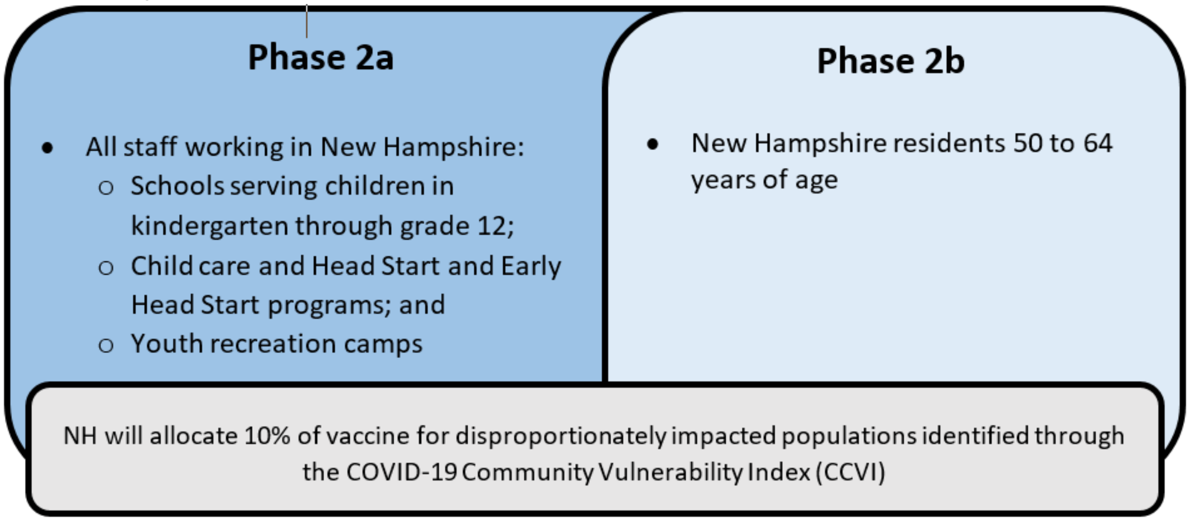

Phase 3a, will include persons < 50 years old with at least one condition putting them at moderately increased risk for severe complications and death of COVID-19 (approximately 325,000 persons) and Phase 3b, will include everyone else not vaccinated (approximately 325,000 persons).

SECTION 4: CRITICAL POPULATIONS

A. Critical Population Identification

Some populations ("critical populations") have been disproportionately impacted by COVID-19 and are at increased risk for infection, severe illness, and death. Marginalized groups have been disproportionately impacted by COVID-19, including widening gaps in health access and health outcomes related to COVID-19, as evidenced in our epidemiologic data. Current epidemiologic evidence shows that nationally, COVID-19 disproportionately affects marginalized racial and ethnic groups including Black, Hispanic or Latinx, American Indian and Alaska Native, and Native Hawaiian and Pacific Islander communities. Increased risk of COVID-19 in these communities and others may be tied to social risk that are a direct result of historical systemic inequities (e.g., disproportionate representation in high-risk jobs in essential industries). Advanced age, specific comorbid conditions, and other factors also put individuals and communities at higher risk for severe COVID-19 morbidity and mortality. The expected allocation of vaccines to these populations is outlined in our allocation strategy above. Critical population groups at increased risk of hospitalization and death may include:

- Healthcare personnel
- Long-term care facility residents (e.g., nursing home and assisted living facility residents)
- People with underlying medical conditions that are risk factors for severe COVID-19 illness
- People 65 years of age and older
- People from racial and ethnic minority groups
- People living and working in other congregate settings
- People with disabilities

B. Critical Population Estimation

On an on-going basis, a combination of internal and external stakeholders are meeting and, where possible, comparing data from multiple sources to determine accurate numbers for distribution planning.

C. Communication Coordination for Critical Populations

Weekly calls between the Incident Management Team and internal and external stakeholders, began in September 2020. This provided opportunities to communicate high-level planning details as the Vaccine Operations Section was being developed. The Vaccine Operations Section's Communication Branch has developed a comprehensive communication plan that clearly delineates

methods for communication with various entities, including the general public and partners that serve critical populations.

NH's vaccine communications planning incorporates an equity lens. Methods of communication messaging are focused and culturally sensitive. Within the communications planning process, NH DHHS has adopted recommendations published by NH Hampshire's COVID-19 Equity Response Team including recognition of the necessity of community engagement and targeted, culturally responsive messaging and outreach. Efforts are made to ensure processes are culturally-responsive to the historical impact of public health vaccination interventions on persons of color in the United States. This includes acknowledging the unethical past of scientific experimentation in the U.S. on Black, Hispanic, Indigenous, and incarcerated populations, among others, that has resulted in vaccine hesitancy, fear and distrust of public health/government systems.

D. Equity Considerations for Vaccine Allocation

COVID-19 has had a disproportionate impact on marginalized populations in NH, much like in the rest of the country. NH's Interactive Equity dashboard shows differences in the prevalence and rate of COVID 19 metrics by age, race, ethnicity and gender. As of November 10, 2020, racial and ethnic minorities in New Hampshire experienced 2.8 times the rate of COVID-19 infection, 4.4 times the rate of hospitalization and 1.5 times the mortality rate compared to white NH residents, after adjusting for differences in age distribution (COVID infection, mortality, and hospitalization data is reported to NH DHHS under NH statute 141:C and population data is sourced from Census Bureau's Population Estimate Program 2018). New Hampshire's COVID-19 Equity Response Team finalized a 50-page report further describing the disproportionate impact of the pandemic on marginalized communities, particularly people of color.

The allocation framework adopts recommendations published by NH Hampshire's COVID-19 Equity Response Team including use of disaggregated data; considerations of significant gaps in data; recognition of the necessity of community engagement and targeted, culturally responsive messaging and outreach. Within phases, we apply a vulnerability index (the COVID-19 Community Vulnerability Index (CCVI)) to identify the communities at highest risk for disproportionate impact of COVID-19 in order to assure equitable access to the vaccine in these populations.

The CCVI identifies communities within the context of the COVID-19 pandemic that may be more vulnerable than others due to limited ability to mitigate, treat, and delay transmission of a pandemic disease, and to reduce its economic and social impacts. It combines indicators specific to COVID-19 with the CDC social vulnerability index (SVI), which measures the expected negative impact of disasters of any type. The CCVI is not designed to predict which individuals will become infected with coronavirus – instead, it tells us about the anticipated negative impact at the community level. This helps decision-makers target resources where they are most needed.

In order to mitigate inequities in health care access and health outcomes, NH is identifying and prioritizing populations that are at a high risk of experiencing health disparities tied to race and ethnicity. Based on NASEM recommendations, NH will withhold 10% of the available vaccine supply at the state level for deployment in areas disproportionately impacted by COVID-19. Areas disproportionately impacted by COVID-19 are identified through the CCVI. The vaccine reserve is distributed to census tracts indicated as areas of high vulnerability according to the CCVI. Among these census tracts of high vulnerability (defined as the top 25 percent of the CCVI distribution within the state) vaccine is distributed proportionally according to population. Education efforts for vaccine distribution will advocate for those who are highest risk within these census tracts to self-select to be vaccinated first so that highest risk individuals receive vaccine first.

SECTION 5: COVID-19 PROVIDER RECRUITMENT AND ENROLLMENT

A. Vaccination Provider Recruitment and Enrollment

Significant planning has been carried out around recruiting and enrolling COVID-19 vaccination providers, including the specific types of settings vaccine is expected to be distributed in for each phase of the initiative. As a universal state for childhood vaccines, New Hampshire already has many providers enrolled in the immunization program. Enrollment will need to be significantly expanded, and will be driven based on the phased vaccine allocation strategy. Instructions for healthcare organizations to enroll as a COVID-19 vaccine provider have been posted online.

B. Provider Types and Settings

A Government/Non-government partnership of distribution is being implemented. This is allows the greatest access to vaccine. By distributing the work between both, we can utilize current, established methods of distribution, such as hospital organizations, provider offices and pharmacies, etc. to offer vaccine to the greatest number of people. This enables the public side, for those that are unable to have medical direction or skilled personnel, to administer vaccine.

Government response:

- The state's two local health departments and 13 RPHNs are disseminating vaccines through closed point of dispensing (POD) agreements. The RPHNs have closed POD plans in place in each region. RPHNs worked with EMS/Fire to vaccinate first responders, who were also able to access vaccine at the state-managed fixed sites if needed.
- The local health departments and RPHNs are also conducting open PODs for vulnerable areas of the population that will have difficulty accessing vaccine through other efforts.
- NH DHHS is operating state-managed fixed sites to provide vaccinations in support of the goal to get individuals vaccinated as quickly as possible.
    - NH DHHS has released detailed guidelines to support operation of the state-managed fixed sites, which may be referenced by other entities planning mass vaccination clinics.
    - Additional public vaccination sites are operated by hospitals and other partners.
    - These public sites operate at variable days and times. Persons scheduling vaccination at one of these sites are able to access the schedule and location details during the scheduling process. These locations are subject to change and individuals should always look to the most up to date information received through the scheduling process.

Non-Government response:

- Hospital networks have been provided vaccine to vaccinate their workforce. Some have also opened public vaccination sites and will eventually vaccinate their own patient populations as more vaccine becomes available.
- Pharmacists and/or other primary vaccination providers may be asked to vaccinate when enough vaccine becomes available to supply to them.
- Healthcare provider offices will also be asked to vaccinate their patients when enough vaccine becomes available to provide to them.
    - If an individual does not have a healthcare provider (i.e. persons experiencing homelessness), they should they be directed to:
        - Local health departments and RPHNs
        - State-managed fixed sites

C. Provider Enrollment Data Collection

Provider enrollment data are submitted via CDC's Vaccine Tracking System (VTrckS) and sent to CDC. Vaccine providers are verified through the established, Vaccine Provider Agreement process, a process that is conducted on an annual basis for all New Hampshire vaccine providers.

D. Provider Credential Verification

Planning efforts currently underway includes work to establish processes to verify that providers are credentialed with active, valid licenses to possess and administer vaccine. This includes working with the existing volunteer registration and credentialing systems in place in New Hampshire for emergency response as well as working with professional boards to access licensing databases. Persons who want to volunteer in the vaccination initiative can register at https://nhresponds.org.

E. Provider Training

Training is accomplished via interactive, on-line, independent learning. CDC TRAIN is being leveraged to provide specific course work for the following topics: PPE; Vaccine Storage & Handling; Clinical Operations (Just-In-Time training); COVID-19 safety measures related to social distancing, and mobile clinics.

F. Vaccine Redistribution

Depending on vaccine availability, doses packaged in larger quantities are delivered to NH DHHS for redistribution. Vaccine accountability staff will facilitate receipt, packaging and redistribution of vaccine doses, observing all relevant cold-chain requirements for individual vaccine presentations.

G. Equitable Vaccine Allocation

Ensuring equitable access to COVID-19 vaccine is central to New Hampshire's vaccine planning efforts. Through established communication methods in previous efforts, continuous external stakeholder feedback, along with current expert planning, will assure equitable distribution of COVID-19 vaccine. The Vaccine Allocation Strategy Branch oversees the allocation plan. This includes soliciting feedback from the SDMAC and feedback from the DHHS Office of Health Equity and the DPHS Equity subject matter expert who sits on the COVID-19 Incident Management Team. Key documents leveraged for this work include New Hampshire's COVID-19 Equity Response Team report, CDC's COVID-19 Response Health Equity Strategy, the National Academies for Science, Engineering, and Medicine's *A Framework for Equitable Allocation of Vaccine for the Novel Coronavirus*, and the CDC's Advisory Committee on Immunization Practices' recommendations. In accordance with these recommendations, beginning in Phase 1b, NH will allocate 10% of the available state vaccine supply to geographic areas that are highly vulnerable to COVID-19. DPHS will:

- Identify communities through the COVID-19 Community Vulnerability Index (CCVI) and US Census data
- Distribute vaccines predominately through mobile vaccination clinic sites
- Coordinate this distribution through the RPHNs at sites that are (1) familiar and accessible to the target population and (2) geographically positioned for easy access on foot or through public transportation when available.
- Initially provide vaccine to NH's racial and/or ethnic minority community then include other vulnerable populations, such as those that are geographically isolated or those living in economic hardship.
- Reserve vaccine for use in targeted response in these identified census tract areas if needed.

H. Pharmacy Participation

All pharmacy distribution plans are reviewed by the NH Immunization Program/Vaccine Operations Section to ensure equitable and fair distribution, based on the Vaccine Allocation Strategy Branch recommendations. Recruitment is through associations, external partners and direct contact to both the commercial retail, private, and independent retail pharmacies. Hospital pharmacies are coordinated through hospital organizations. NH is also working collaboratively with pharmacies enrolled in the federal Pharmacy Partnership LTCF and Retail Programs.

SECTION 6: COVID-19 VACCINE ADMINISTRATION CAPACITY

A. Estimated Vaccine Administration Capacity

Capacity plans were expanded from previous pod exercise experience and plans. Specifically, school-based flu vaccine clinics, recent Hepatitis A vaccine clinics and H1N1 vaccine clinics. Efforts to expand capacity focus on a model of approximately 25-30% of administered vaccine being distributed through the thirteen RPHNs, either through RPHN-run clinics or through the state-run fixed sites. The RPHNs will operate one mobile team in each region. A workforce branch is activated within the Vaccine Operations Section to coordinate state-wide volunteers. Additional efforts to expand capacity include leveraging Emergency Medical Services, a centralized system in New Hampshire, local National Guard, and working with professional medical, nursing, and pharmacy licensing boards and schools to provide vaccinators. This is being built off the foundation of the Medical Reserve Corp.

B. Provider Recruitment Plans

The recruitment plan for vaccinators is informed by the Vaccine Allocation Strategy Branch's final recommendations on phases of vaccine implementation. It is generally recognized that the greatest amount of vaccine providers will be utilized in Phases 2 and forward. The goal is to create vaccine through-put of approximately 100 vaccinations per hour at each of the thirteen fixed locations, while simultaneously providing mobile vaccination clinics to smaller, vulnerable populations that may have more difficulty accessing fixed sites.

On December 22, 2021, a health alert network message was issued that provided instructions to healthcare organizations for how to enroll as a COVID-19 Vaccine Provider. These instructions are also available online. Agreements submitted are reviewed and placed in a queue based on the demographics/risk groups that the provider organization serves and relevance to the current phase of vaccine distribution. Training requirements and information are communicated after the agreement is received and reviewed prior to vaccine distribution. Note that NH DHHS can only provide vaccine to these entities when vaccine supply allows.

SECTION 7: COVID-19 VACCINE ALLOCATION, ORDERING, DISTRIBUTION, AND INVENTORY MANAGEMENT

A. *Allocation Method and Plan*

Current state-wide epidemiology trends inform distribution patterns for priority populations. The goal is to vaccinate one phase of the population throughout the state prior to proceeding with vaccination of the next phase, however, it is understood that due to differences in vaccine uptake in different populations it is more likely that the phases will have overlap as we continue to make vaccine available as quickly as possible. New Hampshire's allocation method has been described earlier. The federal government's allocation plan has assured availability of a second dose of the same vaccine presentation for persons provided their first dose.

B. *Cold Chain Capability*

The different vaccine formulations have various vaccine storage and handling requirements. New Hampshire ensures that all providers receiving vaccine understand the requirements and have the appropriate equipment to ensure proper storage of vaccine. As final storage and handling requirements are released for each vaccine formulation, New Hampshire will determine if additional strategies are needed to provide adequate freezer and deep freezer vaccine storage. Federal supplemental funding has allowed for the purchase of thirteen refrigerators and fourteen freezers. Further funding can be leveraged to assist in purchasing additional equipment if needed. Additional state capacity includes five refrigerators and one freezer. Federal supplemental funding has allowed for the purchase of a pharmacy-grade freezer, one ultra-cold freezer, and forty-two portable refrigerators/freezers (can be used in either capacity). Building upon the knowledge gained as a Universal Vaccine Purchase state, current capacities are measured and reported for the majority of pediatric and family practice clinics. The capacity among these clinics is currently stronger in refrigeration vs freezing capability.

C. *Vaccine Ordering*

Vaccine allocation occurs through vaccine provider agreements under the direction of the NHIP, following the Vaccine Allocation Strategy Branch phases. For Phase 1, New Hampshire used CDC's Vaccine Administration Management System (VAMS) to order vaccine. New Hampshire's Immunization Information System (NHIIS) that will be used to order vaccines for Phase 2 and

beyond. Redundancies have been explored to ensure efficient vaccine ordering and distribution processes. Final vaccine ordering data will reside in the IIS.

D. *Vaccine Repositioning*

Vaccine doses are packaged for shipment from the federal distributor with a minimum quantity requirement (e.g. 100 doses for Moderna, ~1,000 doses for Pfizer-BioNTech). To accommodate smaller orders, NH DHHS receives some shipments of vaccine that can be broken down into smaller allotments and shipped to organizations ordering fewer doses of vaccine. Redistribution of COVID-19 vaccine is completed under the direction of the Vaccine Distribution Branch, under the Vaccine Operations Section. Strike teams of trained vaccine storage and handling personnel are leveraged in the event redistribution of vaccine is required.

E. Vaccine Wastage and Inventory Monitoring

Historically, New Hampshire vaccine wastage is well below CDC's target (<5%) for jurisdictions receiving federally supplied vaccine. Vaccine wastage and inventory reports for COVID-19 vaccine follows established processes for other federally-supplied vaccines, including providing a monthly report. Vaccine wastage and inventory levels have been monitored initially through VAMS and later will be monitored through the NHIIS. Redundancies have been explored to ensure capability for inventory monitoring. Strategies are in place to limit wastage in the field.

SECTION 8: COVID-19 VACCINE STORAGE AND HANDLING

A. Monitoring Adherence to Vaccine Storage and Handling Requirements

As a Universal Vaccine Purchase state, a large number of our Family Practice providers are enrolled with the NHIP. On an annual basis, these practices receive vaccine management training and education. Prior to the receipt of COVID-19 vaccine, new vaccine providers are required to complete vaccine storage and handling training. This training includes mobile clinic vaccine management, in addition to other clinical operation instruction (PPE, COVID-19 screening, drive through clinic considerations and documentation). The training is conducted via an independent, on-line education portal (CDC TRAIN) and followed up with webinars, an established Help Line to include clinical and vaccine management subject-matter experts, and various job aids.

B. Assessing provider/redistribution depot COVID-19 vaccine storage and temperature monitoring capabilities

COVID-19 vaccine storage and handling capabilities are evaluated via the COVID-19 specific, CDC Vaccine Provider Agreement and Profile. Monthly, temperature logs are submitted from all enrolled vaccine providers, and reviewed by NHIP Vaccine Accountability staff following established NHIP processes.

SECTION 9: VACCINE ADMINISTRATION DOCUMENTATION AND REPORTING

A. Vaccine Doses Administered Data Collection

In Phase 1 of vaccine distribution, CDC's VAMs was used to collect data on vaccine doses administered. In future phases, NH DHHS will implement the Vaccine and Immunization Network Interface (VINI) system, developed by NH DHHS through a vendor, for providers who don't have a patient scheduling and documentation software or an Electronic Health Record (HER). If a provider prefers to use their EHR, then they are required to send a flat file to NHIIS daily to report administered doses. Final vaccine administration data will reside in the NHIIS.

The CDC and FDA currently require states to collect certain information for every person who receives a COVID-19 vaccine. The CDC and FDA have stated two primary reasons for these requirements. First, some of the COVID-19 vaccines require two doses, so states need to keep track of individuals receiving vaccinations to ensure each person that receives an initial dose receives the second dose of the same vaccine on the proper timeline. The second reason is that these vaccines have been approved through an emergency use authorization (EUA). This is an unprecedented situation because these are the first vaccines approved through an EUA that are also being distributed nationwide for a mass vaccination. States are therefore being required to keep track of who is getting each vaccine to monitor vaccine safety and efficacy. To bring the State into compliance with Federal requirements, on December 11, 2020, Governor Christopher T. Sununu issued Emergency Order #76, which requires that all patient level information relating to COVID-19 vaccine administration be stored in the NHIIS. The order also temporarily suspends an individual's right to opt-out or withdraw from NHIIS, for COVID-19 vaccine information only. New Hampshire designed its order narrowly to come into compliance with Federal requirements while ensuring that immunization information is kept confidential and stored securely. This order pertains only to sharing of vaccine information and is not related in any way to someone's right to refuse COVID-19 vaccination. NH DHHS has issued FAQs for the public regarding the NHIIS and data privacy.

B. *Submission of Vaccine Administration Data to the Immunization (IZ) Gateway.*

The IZ Gateway is a federal mechanism for connecting IISs or other documentation systems to CDC to share authorized vaccination information. Currently, New Hampshire is scheduled in the fifth phase of an IZ Gateway connection. In the event the IZ Gateway cannot be utilized to provide data to federal partners, New Hampshire will use a manual data upload for reporting purposes.

C. Ensuring COVID-19 Vaccination Provider Readiness for Data Reporting

To ensure real-time documentation and reporting of COVID-19 vaccine administration data from healthcare provider settings, the new state-wide VINI system will be used for providers who can't use their EHR for patient scheduling and will generate a flat file to provide data to the NHIP. Every effort will be made to submit data concurrent with state law, in a timely fashion.

D. Ensuring COVID-19 Vaccination Clinic Readiness for Data Reporting

To ensure real-time documentation and reporting of COVID-19 vaccine administration data from satellite, temporary, or off-site clinic settings, state-wide use of VAMS was implemented for Phase 1 to provide data to the NHIP. Every effort will be made to submit data concurrent with state law in a timely fashion. For Phase 2 and beyond, VINI will be offered to all participating providers in NH.

E. Provider-level Data Monitoring

To monitor provider-level vaccination data to ensure each dose of COVID-19 vaccine administered is fully documented and reported every 24 hours, state-wide use of VAMs was implemented to provide data to the NHIP in Phase 1. Every effort will be made to submit data concurrent with state law in a timely fashion. NHIP will follow up with vaccine providers that do not adhere to vaccine documentation and reporting requirements. For later phases, VINI will be used for providers who can't send a flat file to NHIIS. If a provider is able to generate a flat file and upload it in NHIIS, they are able to use their Electronic Health Records (EHRs) for documentation and monitoring.

F. COVID-19 Vaccination Coverage Reports

COVID-19 vaccination coverage reports will be generated via NHIIS. The reports will be used to identify gaps in vaccine coverage and potential barriers to receipt of vaccine throughout the state. As NHIIS is currently being implemented, a back-up plan of allowing vaccine providers to document in their EHR and upload data via "flat file" directly into the NHIIS will prevent double documentation by facilities that have this capability. This process would require the "flat file" to be uploaded quickly enough to meet the reporting requirements of both the State of New Hampshire and CDC. The advantage of this strategy would be that vaccinators would not need to be trained in an additional system. However, it increases the amount of personnel needed to be assigned to NHIP to monitor and track data quality. New Hampshire releases vaccination data publicly through the department's COVID-19 dashboard and also through periodic reports in the Department's daily updates.

SECTION 10: COVID-19 VACCINATION SECOND-DOSE REMINDERS

A. Methods for Second-Dose Reminders

It is expected that COVID-19 vaccine recipients will need to be reminded of the need for a second dose, should a second dose be required for the vaccine formulation administered. Individuals will be notified when they are eligible for their second dose of COVID-19 vaccine via the NHIIS's reminder/recall feature; through CDC "shot cards" provided upon initial vaccine receipt; or through their medical home's reminder/recall process. Additional options are being explored for second dose reminders to individual patients, for example, reminders via text messaging. Patients that were enrolled and scheduled through VAMS or VINI, and who authorized additional communication, will receive reminders through this system and those enrolled in v-safe will receive notifications through that system as well.

SECTION 11: COVID-19 REQUIREMENTS FOR IISs OR OTHER EXTERNAL SYSTEMS

A. Vaccine Documentation for High-Volume or Temporary Settings

For mobile and off-site vaccine clinics (closed PODs), vaccine administration will be documented via VAMS or VINI or Mobile WebIZ (a standalone documentation system, part of the NHIIS), direct data entry into the NHIIS and redundancies explained below. Vaccinators will utilize tablets, laptops and portable internet connections. Efforts are made to capture patient demographic data prior to clinic start to improve the throughput of patients. Contingency planning for network outages or other access issues includes use of paper documentation if absolutely necessary.

B. Data Collection Elements

It is important to capture information for persons who receive COVID-19 vaccine in order to monitor equitable vaccine distribution and progress towards the vaccination plan's goals. Patient data elements collected include: Name, date of birth, address, contact information, race/ethnicity, allergies/contraindications/precautions, required vaccine information, insurance status. The NHIIS does not have functionality that provides for the documentation of co-morbidities.

C. Data Exchange

Currently, our NHIIS does not accept HL7 messages. If a data use agreement can be established between IZ Gateway and NH's IIS, that HL7 messaging pathway will be prioritized. Additional HL7 onboarding will in the second quarter of 2021.

D. Enrollment in the IIS

Vaccine providers are being rapidly enrolled and onboarded to the NHIIS. Providers complete the CDC fillable pdf enrollment form and submit to NHIP via email; data are then entered manually into the NHIIS or VTrckS. New Hampshire is exploring a second option using a technology-based solution to streamline vaccine provider enrollment.

E. Use of IZ Gateway Connect

The IZ Gateway is a federal mechanism for connecting IISs or other documentation systems to CDC to share authorized vaccination information. The IZ Gateway contributes to a coordinated COVID-19 vaccination response by streamlining these connections and ensuring more up-to-date exchange of immunization data. IZ Gateway connections with New Hampshire are currently being explored. Data use agreement approval will be dependent upon CDC clarification related to data element requirements, as well as CDC's prioritization of New Hampshire's connection to the IZ Gateway.

SECTION 12: COVID-19 VACCINATION PROGRAM COMMUNICATION

A. COVID-19 Vaccination Communication Plan

New Hampshire's COVID-19 vaccination communication plan is in place and will continuously be updated to include addressing communications with key audiences, identification of effective communication channels, and partner activation for each of the phases of the COVID-19 Vaccination Program. A Communication Branch was formed as one of seven branches in the Vaccine Operations Section, within the Incident Management Team. Implementation of the communications plan is ongoing; it will include the identification and active engagement of internal partners and external stakeholders in the planning process. Key internal partners include subject-matter experts in infectious disease/epidemiology, vaccine management, communications, representation from housing, equity groups, Emergency Medical Services and the Emergency Services Unit. External partners include long-term care and assisted living facilities, hospital organizations (which also represent a large number of health care practices), schools, retail, private and hospital pharmacies, health care coalitions and associations, and professional medical societies.

New Hampshire has established a dedicated COVID-19 website where information is posted for partners and the public. This includes information for healthcare providers as well as FAQs for various audiences and other information related to New Hampshire's COVID-19 Vaccine Plan. Additionally, 2-1-1 has been expanded to support vaccine–related calls and vaccine scheduling for persons who do not have access to internet or otherwise need assistance.

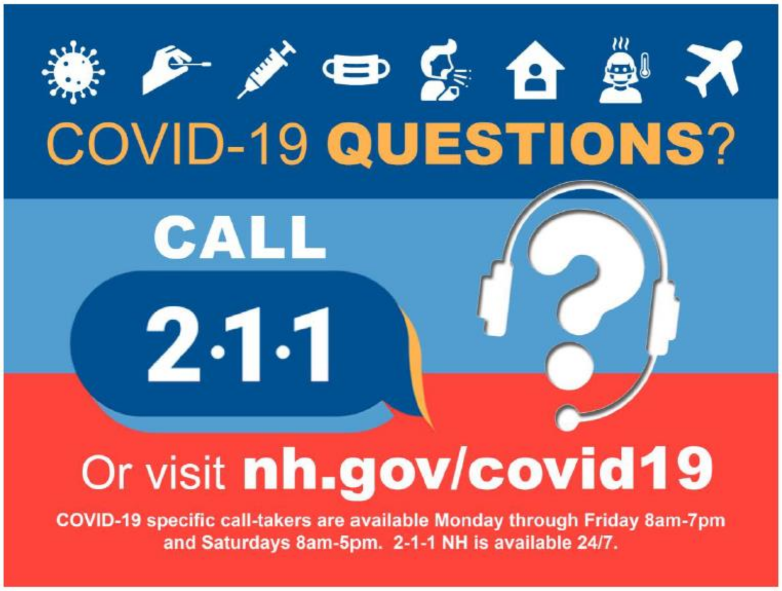

B. Crisis and Emergency Risk Communication

NH DHHS has established processes for providing crisis and emergency risk communications in an expedited manner. This includes leveraging the State of New Hampshire's Joint Information Center. For clinical or partner messaging, subject-matter experts are consulted in the development of communications, which are provided through Health Alerts via the Health Alert Network (HAN) and which reach >14,000 healthcare providers and other public health partners in New Hampshire. For public messaging, a variety of platforms are used to push information to the public. A member of the Department's Public Information Office leads the vaccine planning Communication Branch, which allows for flow of information and consistent messaging across the COVID-19 response.

SECTION 13: REGULATORY CONSIDERATIONS FOR COVID-19 VACCINATION

A. Emergency Use Authorization and Vaccine Information Statement Provider Education

Ensuring enrolled COVID-19 vaccination providers are aware of, know where to locate, and understand the information in any Emergency Use Authorization (EUA) fact sheets for providers and vaccine recipients will be important. The Communication Branch will provide EUA fact sheets for vaccine providers and vaccine recipients through the COVID-19 website, social media platforms, links to the CDC website and direct email to enrolled providers. Information is also distributed via Health Alerts. CDC also maintains a website for posting the most up-to-date information on COVID-19 vaccines as they become available.

B. Emergency Use Authorization and Vaccine Information Statement Distribution

New Hampshire will follow guidelines and recommendations provided through the CDC as it relates to the distribution of fact sheets under a EUA and will distribute this information to healthcare providers in New Hampshire through Health Alerts and calls.

Pfizer Medical Information Line	1-800-438-1985<br><br>Pfizer: https://www.cvdvaccine-us.com/<br>CDC: https://www.cdc.gov/vaccines/covid-19/info-by-product/pfizer/index.html<br>FDA: https://www.fda.gov/emergency-preparedness-and-response/coronavirus-disease-2019-covid-19/pfizer-biontech-covid-19-vaccine
Moderna Call Center<br>Available 8am to 8pm EST, Monday through Friday. We are here to support you as you begin vaccinations and can assist in answering questions in real-time. Please do not hesitate to reach out if you or someone you know needs assistance or has other inquiries."	1-866-MODERNA (1-866-663-3762)<br><br>Moderna:<br>https://www.modernatx.com/covid19vaccine-eua/<br>CDC: https://www.cdc.gov/vaccines/covid-19/info-by-product/moderna/index.html<br>FDA: https://www.fda.gov/emergency-preparedness-and-response/coronavirus-disease-2019-covid-19/moderna-covid-19-vaccine

Janssen Biotech	1-800-565-4008<br><br>Janssen Biotech: http://www.janssencovid19vaccine.com/<br>CDC: https://www.cdc.gov/vaccines/covid-19/info-by-product/janssen/index.html<br>FDA: https://www.fda.gov/emergency-preparedness-and-response/coronavirus-disease-2019-covid-19/janssen-covid-19-vaccine

SECTION 14: COVID-19 VACCINE SAFETY MONITORING

A. Reporting of Adverse Events to the Vaccine Adverse Event Reporting System (VAERS)

New Hampshire will ensure enrolled COVID-19 vaccination providers understand the requirement and process for reporting adverse events following vaccination to VAERS. Through the vaccine provider enrollment process, COVID-19 vaccine providers will receive information related to the requirement and process for reporting adverse events to VAERS. Information will also be made available through the COVID-19 website, social media platforms and quarterly Immunization program conference calls.

Patients also have the opportunity to enroll in v-safe to provide information on expected vaccine side effects that would not be reported in VAERS. V-safe is a smartphone-based tool that uses text messaging and web surveys to provide personalized health check-ins after a vaccine recipient receives a COVID-19 vaccination. Through v-safe, the patient can tell CDC if they have any side effects after getting the COVID-19 vaccine. v-safe also reminds the patient to get their second COVID-19 vaccine dose if they need one. NH DHHS does not have access to v-safe information; the system is managed by CDC. NH DHHS encourages people in New Hampshire to participate in v-safe after receiving the vaccine to help CDC monitor vaccine safety.

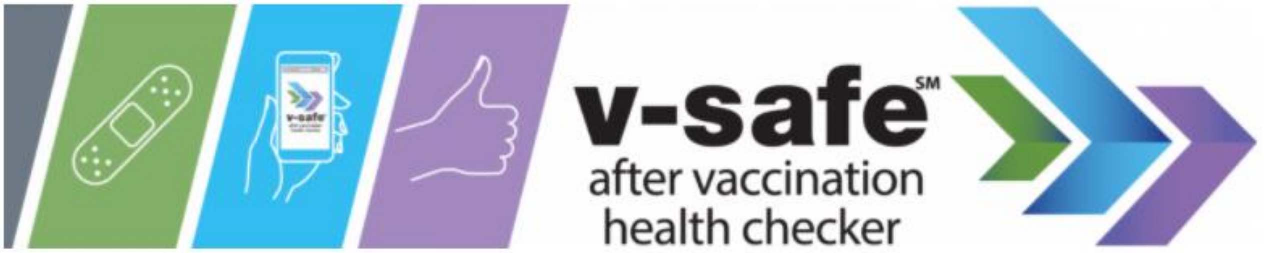

SECTION 15: COVID-19 VACCINATION PROGRAM MONITORING

A. Program Progress Reporting:

New Hampshire will implement methods and procedures for monitoring progress in COVID-19 Vaccination Program implementation, including monitoring:

- Provider enrollment
- Access to COVID-19 vaccination services by populations in all phases of implementation
- IIS or other designated system performance
- Data reporting to CDC
- Provider-level data reporting
- Vaccine ordering and distribution
- 1- and 2-dose COVID-19 vaccination coverage

Vaccine Agreements are executed using the following methodology. Providers identified as vaccinators in the first phase were prioritized for enrollment. After enrolling these vaccine providers, additional providers were recruited based on utilization in the next phases, as well as creating depth of vaccine providers and decreasing barriers towards immunization. This is achieved by reviewing geographic and population base data for the state. Providers unable to vaccinate will be assessed on an individual basis, and opportunities to provide vaccination in their area will be explored. Additionally, fixed sites stood up on the government side of the response were established to back up these individual vaccine providers. Pharmacies were also recruited to provide additional vaccination services.

Vaccine providers are required to document COVID-19 vaccine in the current COVID-19 documentation system. Those unwilling to comply will not be shipped additional vaccine and additional attempts will be made to establish new vaccine providers in the area. Recognizing the burden of documentation (entry into their own EMR and in the state's documentation system) NHIP is making every effort to explore all options for large data migrations from existing electronic health record systems, compliant with state regulations. Vaccine ordering and distribution is approved at the state level and vaccine wastage is monitored through standard, established procedures. State-wide documentation will be leveraged to ensure the second dose of a vaccine presentation is the same as the first, observing recommended intervals between vaccine doses.

B. Resource Monitoring

Methods and procedures for monitoring resources are in place, including monitoring the budget, staffing, and supplies. All resource management for COVID-19 vaccine distribution is currently being managed by NHIP as part of the Vaccine Operations Section. This same methodology will be applied to future methods and procedures for monitoring resources. The Vaccine Operations Section will also leverage resources within the larger Incident Management Team structure, which includes Finance, Planning, and Logistics sections.

C. Communication Monitoring

The Communications Branch, established under the Vaccine Operations Section, utilizes communication subject-matter experts to create, inform and standardize communication pathways. Future plans will address implementing communication strategy evaluation methods, to include monitoring the success of message delivery and message reception among target audiences. Currently, communication has been broken out into sub categories: RPHNs, external health care provider entities/organizations, national, corporate vaccine providers, contracted through the CDC, and the general public. Reception of partner communication messages will be monitored through bidirectional communication with stakeholder groups. Reception of public communication messages will be monitored through the joint information center, the NH DHHS public information office, media inquiries, and social media monitoring.

D. Local-level Situational Awareness Monitoring

Bi-directional communication pathways have been established and leveraged to provide real-time feedback to inform New Hampshire's COVID-19 Vaccine response efforts. Some examples of this include, Chief Information Officers of large hospital organizations; professional medical societies in New Hampshire, Granite State Health Care Coalition, NH Hospital Association, RPHNs, LTCF through the New Hampshire Healthcare Association, and many others.

E. Vaccination Program Metrics

Program metrics to be monitored include vaccination provider enrollment, doses distributed, doses administered, and vaccination coverage. Additional metrics may be developed. Relevant COVID-19 vaccine data have been incorporated into the Department's COVID-19 dashboard to provide vaccine-specific information to the general public and state and local officials. Further reporting will be generated as needed.

APPENDIX 1: INCIDENT MANAGEMENT TEAM STRUCTURE

This organizational chart reflects the COVID-19 public health response organizational structure. There are three Operations Sections that report to the Incident Commander but are not depicted in this chart. These three Operations Sections are the primary operational units in the public health response and include: 1.) Case Investigation, Contact Tracing, and Epidemiology and Surveillance, 2.) Testing, and 3.) Vaccine Planning.

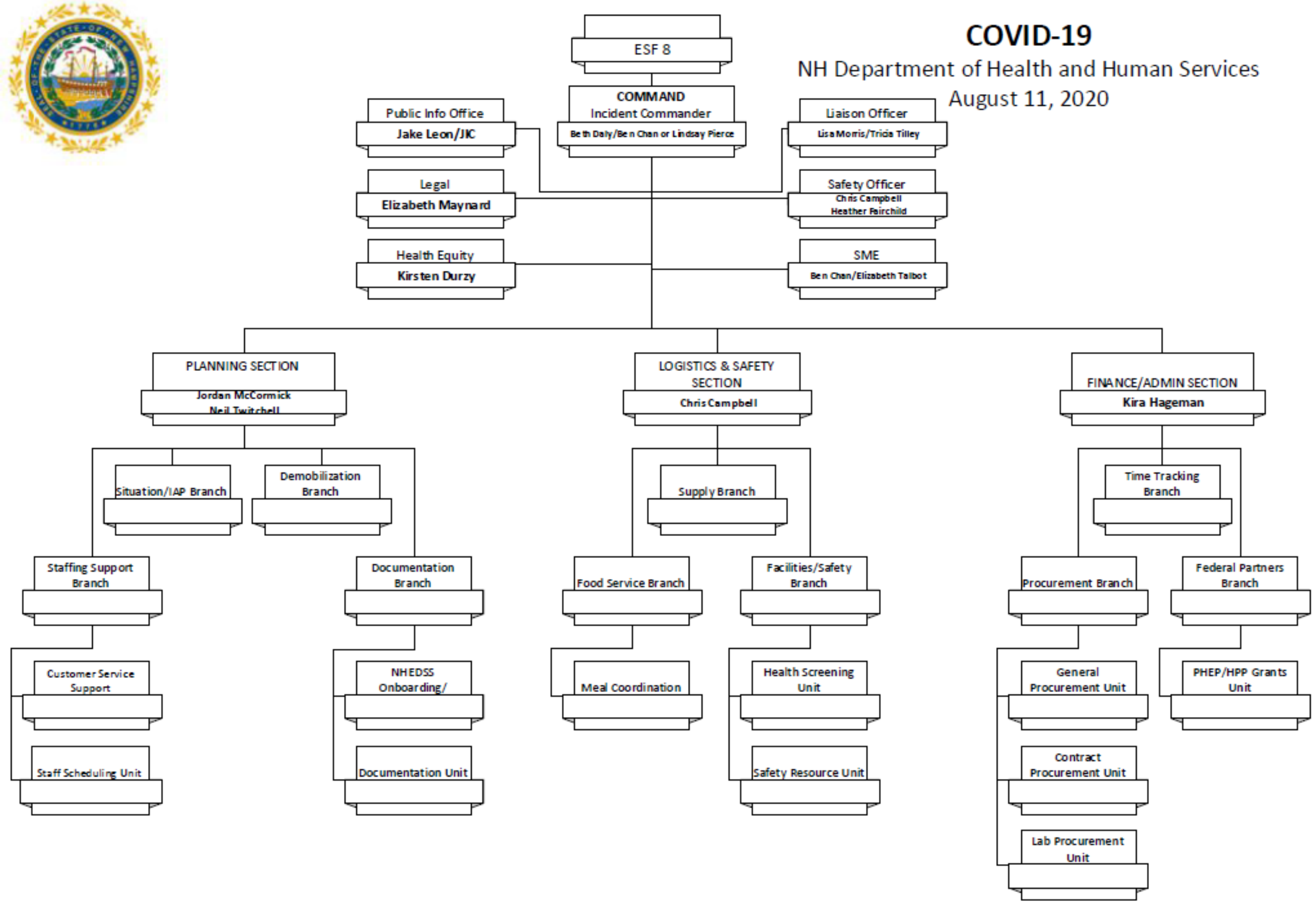

APPENDIX 2: VACCINE OPERATIONS SECTION

This organizational chart reflects the COVID-19 vaccine planning organizational structure.

**Incident Commander**: Dr. Beth Daly	
**Vaccine Operations Section** Chief: LCDR Torane Hull, Deputy: Colleen Haggerty, Support: Elinor Fenton	
**Vaccine Allocation Strategy Branch**<br>Director: Dr. Elizabeth Talbot<br>Deputy: TBD<br>SDMAC* Liaison:<br>Dr. Jonathan Ballard	• Develop vaccine allocation strategy with regard to clinical, ethics, and equity considerations<br>• Estimate phase group populations
**Communications Branch**<br>Director: Laura Montenegro, PIO<br>Deputy: TBD	External Partners<br>• RPHNs<br>• Healthcare (e.g. LTCFs, hospitals, provider practices, EMS, etc.)<br><br>General Population<br>• Communicate strategy<br>• General Inquiries<br>• Information on where to get vaccinated<br>• Why should people get vaccinated
**Vaccine Storage and Handling Branch**<br>Director: Lena Boulanger<br>Deputy: Rachel Moon	• Order and Distribution Approval<br>• Shipping/Number gathering<br>• Vaccine storage and handling training:<br>o RPHNs<br>o Healthcare Providers<br>• Vaccine storage and handling at Depot<br>o Repackaging for orders under 100<br>o Vaccine redistribution<br>• Vaccine agreement- for participating HCP
**Logistics Branch**<br>Director: Craig Beaulac<br>Deputy: Sean Heichlinger	• Non-vaccine storage and inventory<br>• Healthcare Provider Equipment distribution as able<br>• RPHN equipment and supply allocation
**Workforce Branch**<br>Director: Fallon Reed<br>Deputy: TBD	• Planning functions to fulfill workforce needs<br>• Identifying and acquiring staffing resources
**Documentation Branch**<br>Director: Alok Patra<br>Deputy: Jackie Ramirez	Documentation /Ordering Training<br>• IIS Training:<br>o General Providers<br>o RPHN-Mobile Web IZ<br>Backup documentation methods<br>• VAMs<br>• State created solution<br>Documentation Support<br>• Helpdesk<br>Educational webinars

**Medical Direction Branch**<br>Director: Dr. Ben Chan<br>Deputy: Janice Houston	• SOP and Clinical Direction Creation<br>• Nurse RPHN Liaison<br>o 1:2 support**<br>o Vaccination questions and support<br>• RPHN Medical Direction Training:<br>o Just In Time Training<br>o PPE training<br>o COVID Screening Training<br>Alternate Clinic site training
Vaccine Allocation Strategy Participants: Dr. Elizabeth Talbot, Dr. Jonathan Ballard, Maj. Lyndsey Fleming, Incident Commander, Vaccine Operations Section Chief and Deputy, Branch Leads, HSEM, Dr. Ben Chan, Kirsten Durzy, Tricia Tilley, Lisa Morris, Neil Twitchell, Stephanie Locke, Melissa Hatfield,<br>*The State Disaster Medical Advisory Committee will be consulted in an advisory capacity.	

APPENDIX 3: IMMUNIZATION PROGRAM SECTION

This organizational chart reflects the day-to-day organizational structure of the Immunization Program Section irrespective of COVID-19 planning.

New Hampshire Department of Health and Human Services
Division of Public Health Services
Bureau of Infectious Disease Control
Immunization Program Section

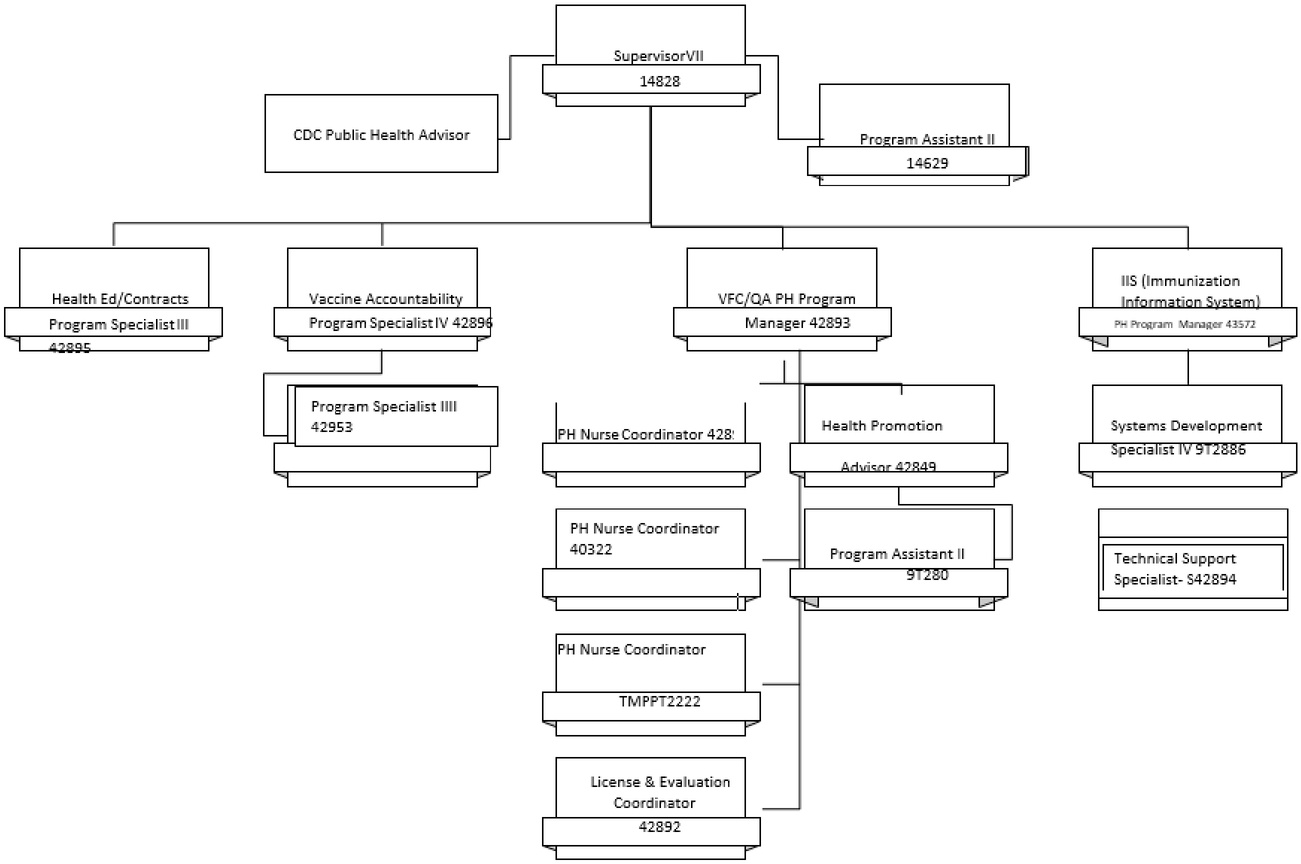

APPENDIX 4: STATE DISASTER MEDICAL ADVISORY COMMITTEE (SDMAC)

- Jonathan Ballard, MD, MPH, MPhil – NH Department of Health and Human Services, Chief Medical Officer
- Kathy A. Bizarro-Thunberg, MBA, FACHE – Executive Vice President / Federal Relations, New Hampshire Hospital Association
- Charles P. Burney, MD – Resident, General Surgery and Leadership Preventive Medicine
- Michael Calderwood, MD – Infectious Disease and International Health, Dartmouth Hitchcock Medical Center
- Rep. Polly Campion, MS, RN – New Hampshire House of Representatives
- Patricia E. Clancy, MD – Pleasant St. Family Medicine, Chair, Concord Hospital Ethics Committee
- Carl Cooley, MD – Developmental Pediatrician
- Adam Crepeau – Policy Director, Office of Governor Chris Sununu
- James Culhane – President and CEO, Home Care Hospice & Palliative Care Alliance
- Kevin P. Desrosiers, MD, MPH – Chief Medical Officer, Elliot Hospital and Elliot Medical Group Acute Care Services, Vice President of Medical Affairs, Physician - Critical Care and Preventive Medicine
- Jeff Dickenson – Advocacy Director, Granite State Independent Living
- Paul Drager, JD – MedEthics Consulting
- Anne Edwards, Esq. – Associate Attorney General, NH Department of Justice
- John E. Friberg, Jr. Esq. – Chief Legal Officer, Manchester and Nashua SolutionHealth
- Marc D. Hiller, MPH, DrPH – Associate Professor, Department of Health Management and Policy, College of Health and Human Services, University of New Hampshire
- Lucy C. Hodder, JD – Director, Health Law and Policy, Professor of Law, University of New Hampshire, Franklin Pierce School of Law, Institute for Health Policy and Practice
- Joseph Hoebeke, Chief, Hollis Police Department
- Sally Kraft, MD, MPH, VP of Population Health at Dartmouth-Hitchcock
- Richard Levitan, MD – Emergency Medicine, Littleton Regional Hospital
- John McAllister, President, Professional Fire Fighters of NH
- Kenneth Norton LICSW – Executive Director, National Alliance of Mental Illness, New Hampshire Chapter
- Debra Pendergast – New Hampshire Department of Safety, Director of the Division of Fire Standards and Training and Emergency Medical Services
- James G. Potter – Executive Vice President/CEO, New Hampshire Medical Society

- Susan A. Reeves, EdD, RN, CENP – Chief Nurse Executive, Dartmouth-Hitchcock Health Executive Vice President, Research & Education, Dartmouth-Hitchcock, Clinical Professor, Department of Community and Family Medicine
- Kate Riddell, MD – Anesthesiologist, Southern New Hampshire Medical Center
- Rae Ritter, MSN, APRN-CRNA – President, New Hampshire Association of Nurse Anesthetists
- Luanne Rogers, RN – Administrator, St. Theresa's Rehabilitation and Nursing Center
- Justin Romello – New Hampshire Department of Safety, Bureau Chief, Division of Fire Standards and Training and EMS
- David Ross, Administrator – Hillsborough County Nursing Home
- Michael Sitar, Jr. Chief, Tilton Northfield Fire and EMS
- Luanne Rogers, RN – Administrator, St. Theresa's Rehabilitation and Nursing Center
- Sedden R. Savage, MD, MS – Addiction Medicine and Pain Medicine Clinician, Advisor to Dartmouth Hitchcock Substance Use and Mental Health Initiative
- Sen. Tom Sherman – New Hampshire Senate
- Steve Surgenor, MD – Anesthesiologist, Dartmouth Hitchcock Medical Center
- Elizabeth Talbot, MD – New Hampshire Department of Health and Human Services, Deputy State Epidemiologist, Dartmouth Hitchcock Medical Center, Infectious Disease Clinician
- Robert Theriault, Jr., BSPharm, MB, RPh – Director of Pharmacy Services, Wentworth-Douglass Hospital
- Joan C. Widmer, MS, MSBA, RN, CEN – Nurse Executive Director, New Hampshire Nurses Association
- Tom Wold, DO – Chief Medical Officer, Portsmouth Regional Hospital
