# Supplementary material for: Ensuring Equitable COVID-19 Vaccine Allocation in New Hampshire: The First Eight Months toward a New Era
Source: Vaccines (Basel). 2022 Aug 29;10(9):1421. doi: 10.3390/vaccines10091421 (PMC9501825; doi:10.3390/vaccines10091421)
Supplement: Supplementary file 1 [file vaccines-10-01421-s001.zip › vaccines-1843494-supplementary/S2.pdf]

NH DIVISION OF Public Health Services
Department of Health and Human Services

Bureau of Infectious Disease Control

New Hampshire COVID-19 Vaccination Allocation Plan Summary
April 6, 2021

Phase 1

Phase 1a (~110,000)
- High-risk health workers
- First responders
- Residents and staff of long-term care and assisted living facilities

Phase 1b (~325,000)
- People ≥65 years old
- Medically vulnerable at ***significantly*** higher risk 2 or more conditions (see list)
  - Family caregivers of those medically vulnerable persons, <16 years old, not eligible for vaccine
- Residents and staff of residential facilities for persons with intellectual and developmental disabilities
- Corrections officers and staff working in correctional facilities
- First responders and health workers not already vaccinated

Phase 2

Phase 2a (~75,000)
- K-12 school and childcare staff

Phase 2b (~200,000)
- People 50 – 64 years old

Phase 3

Phase 3 (~650,000)
- Everyone else not already vaccinated

Estimated Timeframe: DECEMBER - MARCH | MARCH | MARCH AND BEYOND

*** Estimated timeframe depends on vaccine doses allocated to New Hampshire from the federal government and vaccine uptake***

Equity is a crosscutting consideration: Vaccine access will be prioritized for geographic areas identified through the COVID-19 Community Vulnerability Index (CCVI).

List of Underlying Medical Conditions (adapted from CDC):

Phase 1b: Two or more conditions

- Cancer
- Chronic Kidney Disease
- COPD (Chronic Obstructive Pulmonary Disease) and other high-risk pulmonary disease
- Down Syndrome
- Heart Conditions, such as heart failure, coronary artery disease, or cardiomyopathies
- Immunocompromised states
- Obesity (body mass index of 30 kg/m or higher)
- Pregnancy
- Sickle cell disease
- Type 2 Diabetes Mellitus

Note: DPHS allows a health care provider to vaccinate any patient assessed to have significant risk for severe illness due to co-morbidities, even if not listed here. This list does not include every condition that might increase one's risk for developing severe illness from COVID-19, such as those for which evidence may be limited (e.g., rare conditions or combinations of conditions).
