# Supplementary material for: Ensuring Equitable COVID-19 Vaccine Allocation in New Hampshire: The First Eight Months toward a New Era
Source: Vaccines (Basel). 2022 Aug 29;10(9):1421. doi: 10.3390/vaccines10091421 (PMC9501825; doi:10.3390/vaccines10091421)

Guidelines for Vaccine Equity Allocation (VEA)
March 19, 2021

New Hampshire is taking a phased approach to vaccine rollout, targeting vulnerable populations with the initial allotment of vaccines from manufacturers. All phases of vaccine rollout are outlined in a separate document, the NH COVID-19 Vaccination Plan. Beginning in Phase 1b and continuing in each subsequent phase, NH will allocate 10% of vaccine for disproportionately impacted and highly vulnerable populations predominantly identified through the COVID 19 Community Vulnerability Index (CCVI). The purpose of this document is to aid decision making regarding the vaccine allocated for equity. This document is subject to change, as vaccine efficacy and safety data emerge and national guidance evolves. This document provides:

- Considerations for the Vaccine Equity Allocation (VEA)
- Methods for the Vaccine Equity Allocation including eligibility, allocation plan, use of the CCVI, and community partnership

Considerations for Vaccine Equity Allocation

COVID-19 has had a disproportionate impact on certain sub-populations in New Hampshire. These sub-populations are vulnerable for a variety of reasons, some in common and some unique, and as a result experience higher infection rates, are prone to more severe illness, and experience worse outcomes (such as hospitalization and death) than the rest of the population. It is imperative to increase vaccine access to these vulnerable populations by diminishing physical, language, social and transportation barriers for at least four reasons. First and foremost, the NH Vaccine Allocation Plan has a stated overall goal of equity and fairness in vaccine distribution, which includes trying to prevent subpopulations from lagging behind others in the allocation of vaccine because of factors largely beyond their control such as education about the vaccine, access to healthcare or the vaccine registration system and/or distribution sites, higher risk employment, nature of their housing, among others. Second, a key goal of the NH vaccination plan is to reduce mortality so the subpopulations that evidence shows us are most likely to die must be prioritized for protection. Third, if disease transmission is not controlled in all populations in NH (i.e., these vulnerable subpopulations remain susceptible to infection), chains of transmission continue (R_T >1), the COVID-19 epidemic will continue and the health of the population as a whole will remain vulnerable, especially because protection from infection and possibly from immunization is not lifelong. Fourth, the more individuals progress to severe illness, the greater the burden on our healthcare systems. Severe illness, for example, taxes hospital resources such as oxygen, ventilators, specific treatments such as Remdesivir, Regeneron, or monoclonal antibodies, available inpatient beds, but also interrupts routine outpatient and preventive healthcare, as the healthcare system is short of staff, personal protective equipment and necessarily institutes strategies to avoid in-facility viral transmission.

Health Equity and vaccination program implementation are closely linked. The current system in NH of distributing vaccines will work for most but not all people. Successful implementation of the COVID-19 vaccination program and confidence in COVID-19 vaccines are pivotal to reducing existing health inequities related to COVID-19 (CDC ACIP). NH DHHS is committed to ensure equitable access for the most affected communities.

Methods for Vaccine Equity Allocation

Eligibility

Persons are eligible to receive equity doses, regardless of phase of vaccination a person is otherwise identified in, if they:

- Reside in priority locations (see Appendix A) using the COVID-19 Community Vulnerability Index (CCVI) and US Census data 19; and/or
- Meet one or more eligibility criteria regardless of where they live in New Hampshire:
  - Identify as racial and ethnic minorities (all persons except white, non-Hispanic)
  - Currently experiencing homelessness (sheltered and unsheltered)
  - Low income (household income at or below 185% of the poverty level)
  - Geographically isolated or encounter physical or other barriers to travel (i.e., limited access to transportation) to the State points of vaccine distribution
  - Homebound[1]
  - Medically vulnerable and do not have a medical home to verify their conditions
  - Language/communication access barriers that prevents them from understanding registration instructions and assent during the documentation process
  - Other significant barriers that prevent someone from being vaccinated through other State mechanisms

When vaccine supply is limited and circumstances allow, DPHS recommends that administration can be prioritized among vulnerable populations at risk of disproportionate impact from COVID-19. Considerations for prioritization in situations of limited vaccine supply are outlined below:

Vaccinate those who either:	Are 65 years or older before those who are younger
	Have more medical comorbidities before those with fewer
	Live in multi-generational households before those who do not
	Have reduced opportunities for accessing healthcare, transportation or other key supportive services necessary to access vaccination
	Have more public contact than those with less contact
	Without confirmed COVID-19 within the previous 90 days before those with confirmed COVID-19 in the previous 90 days
	Have no opportunity to receive vaccine through other locations or providers before those who do

[1] The reasons for being homebound include: a) The person's doctor believes that their health or illness could get worse if they leave the home; b) The person requires the help of another person and/or medical equipment to leave the home, or find it difficult to leave the home and typically cannot do so. Note being homebound includes going out for medical appointments or treatment or for short periods of time or for special non-medical events.

NH DHHS Vaccine Equity Allocation Plan:

- DHHS will direct up to 10% of vaccine to eligible persons
- Vaccine Equity Branch within DHHS analyzes disaggregated State and national data from multiple sources that informs appropriate targets for distribution
- Distribute vaccine predominantly through the NH Regional Public Health Networks (RPHNs) via mobile vaccination clinic sites
- Implement focused and culturally sensitive methods of communication and outreach in order to engage with eligible persons and address issues of vaccine hesitancy

COVID-19 Community Vulnerability Index:

DHHS identified priority locations using the COVID-19 Community Vulnerability Index (CCVI) and US Census data in order to 1) identify the communities at highest risk for disproportionate impact of COVID-19 and 2) to assure equitable access to the vaccine in these populations. The COVID-19 Community Vulnerability Index (CCVI) identifies communities within the context of the COVID-19 pandemic that may be more vulnerable than others due to limited ability to mitigate, treat, and delay transmission of a pandemic disease, and to reduce its economic and social impacts. It combines indicators specific to COVID-19 with the CDC social vulnerability index (SVI), which measures the expected negative impact of disasters of any type. The CCVI is not designed to predict which individuals will become infected with coronavirus – instead, it tells us about the anticipated negative impact at the community level. This helps decision-makers target resources where they are most needed. CCVI data helps guide location of vaccination efforts.

Community Partnerships & Registration

Community agencies that serve populations outlined in the eligibility criteria above work directly with RPHNs and DHHS to host equity clinics. Agencies that are hosting a clinic will work with RPHNs to register people. DHHS, Regional Public Health Networks, and partnering agencies are doing extensive community outreach in areas and to populations that are identified as vulnerable as described above. Community organizations who are interested in partnering with their RPHN should reach out directly to their local RPHN. Individuals who may be eligible for the equity allocation can call 2-1-1 or email covidvaccine@dhhs.nh.gov.

APPENDIX A: Equity Vaccine Allocation Planning Map

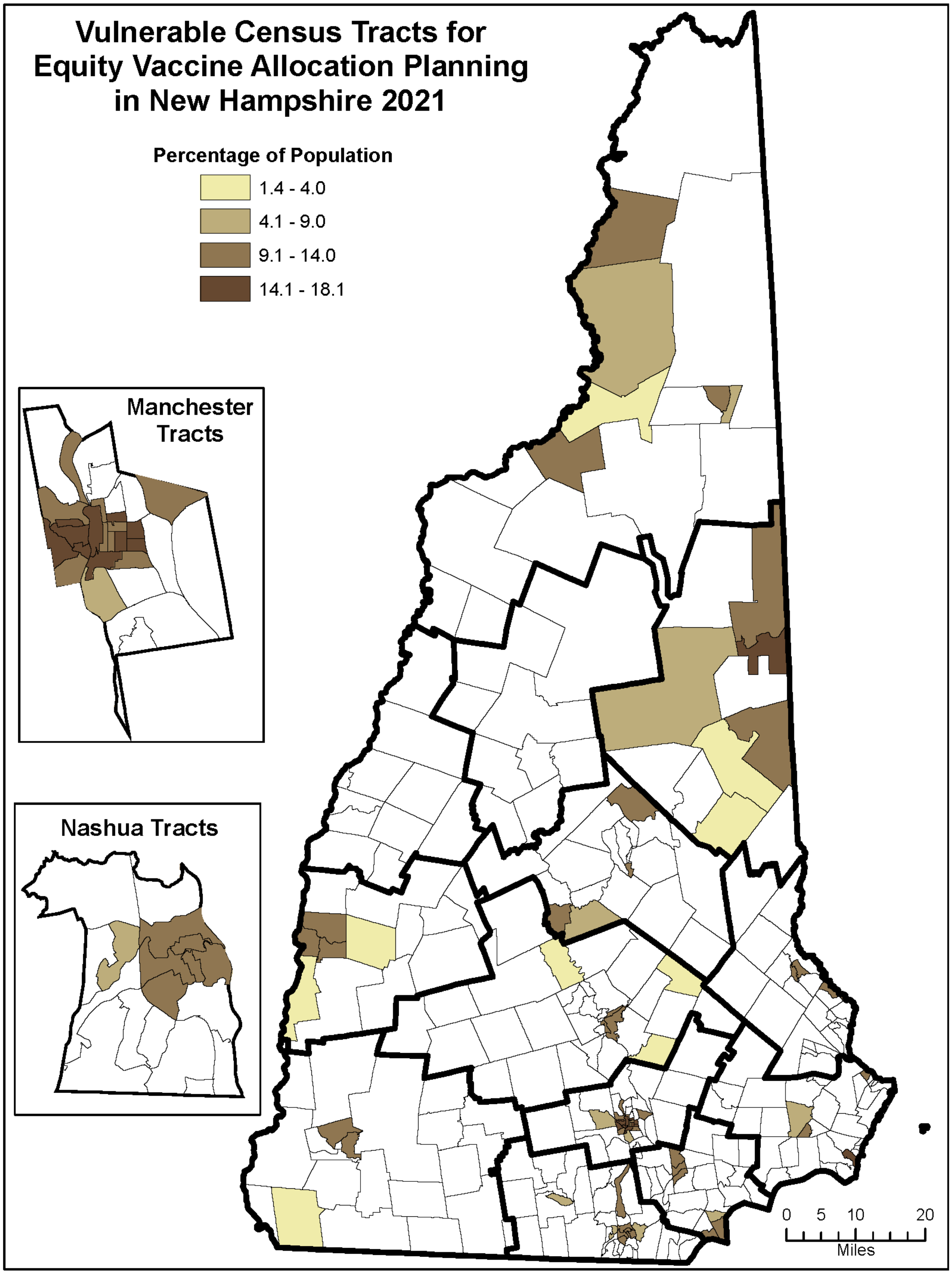

Supplement: Supplementary file 1 [file vaccines-10-01421-s001.zip › vaccines-1843494-supplementary/S3.pdf]
