# Supplementary material for: Ensuring Equitable COVID-19 Vaccine Allocation in New Hampshire: The First Eight Months toward a New Era
Source: Vaccines (Basel). 2022 Aug 29;10(9):1421. doi: 10.3390/vaccines10091421 (PMC9501825; doi:10.3390/vaccines10091421)
Supplement: Supplementary file 1 [file vaccines-10-01421-s001.zip › vaccines-1843494-supplementary/S4.pdf]

Bureau of Infectious Disease Control

NH COVID-19 Vaccination Allocation Guidelines for Phase 1a
February 5, 2021

(changes in **red**)

NH is taking a phased approach to vaccine rollout, targeting critical populations with the initial allotment of vaccines from manufacturers. These critical populations will receive the vaccine during Phase 1a. Subsequent phases are outlined in a separate document, the NH COVID-19 Vaccination Plan. The purpose of this document is to aid decision making regarding Phase 1a populations. Guidance regarding later phases will be released as available. This document is dynamic, and subject to change, as vaccine efficacy and safety data continue to emerge and national guidance evolves. This document provides:

- An overview of the three populations within phase 1a
- Detailed descriptions of populations in New Hampshire's phase 1a allocation plan
- Principles for employers and others involved in vaccine allocation in the event of limited dose availability

Phase 1a: Limited Doses Available, "Jump Start Phase"

The New Hampshire Division of Public Health Services (DPHS) includes the following groups for vaccination under Phase 1a: 1) at-risk health workers in the most and moderate risk groups; 2) older adults in residential care settings; and, 3) first responders. The following graphic is an overview of NH Vaccine Allocation Phase 1a, with additional description of the groups below:

At-Risk Health Workers

Most risk: Front line clinical staff who provide direct patient care and support staff with risk of exposure to bodily fluids or aerosols.

Moderate risk: Staff who have indirect or limited patient contact.

Older Adults in Residential Care Settings

Residents of long term care facilities (LTCF), skilled nursing (SNF), and assisted living facilities (ALF).

First Responders

Fire and EMS: All private, public, and volunteer fire and emergency medical services (EMS) personnel.

Law Enforcement: All certified or sworn law enforcement officers in NH, whether employed full- or part-time.* Includes Federal law enforcement not covered by federal allocation.

COVID-19 Responders: Personnel with the NH COVID response with potential patient or SARS-CoV-2 virus contact, including COVID-19 testing laboratorians and specimen collection personnel.

*Does not include officers working in correctional facilities, which are included in a later phase.

Description of Populations Within Phase 1a

I. At-Risk Health Workers

New Hampshire DHHS includes in Phase 1a of the vaccine allocation strategy at-risk health workers who either:

(1) Work in situations where the risk of SARS-CoV-2 transmission is higher, or

(2) Are at an elevated risk of transmitting infection to patients at higher risk of mortality and severe morbidity.

Consistent with the National Academies of Sciences, Engineering, and Medicine (NASEM), NH defines at-risk health workers as **paid or unpaid** health workers who are **clinicians and other workers** caring for patients **in a variety of settings** including: acute, rehabilitation and psychiatric hospitals; ambulatory and urgent care clinics; dialysis centers; blood, organ, and tissue donation facilities; nursing homes; and other settings (see Table 1). This group will include traditional healthcare roles but also may include others who have received formal specialized medical training that enables them to do clinical care procedures. For example, school paraprofessionals are not routinely included within this definition of health workers unless they have received such specialized training. The person does not need to have earned a clinical degree, but are reasonably expected to have ongoing exposure to patients in the course of their work, including those who distribute or administer the COVID-19 vaccine such as pharmacists; plasma and blood donation workers; public health nurses; COVID-19 laboratorians; and morticians, funeral home workers and other death care professionals.

Table 1: Settings in which Phase 1a health workers work

Setting	Roles[1]
Acute, rehabilitation and psychiatric hospitals	Multiple
Nursing homes, assisted living facilities	Multiple
Ambulatory care settings	
Ambulatory and urgent care clinics	Multiple
Dialysis centers; blood, organ, and tissue donation facilities	Multiple
Other settings	
K-12 schools	School nurse
Home health care	Home health aide, personal care aide, occupational therapist
Funeral services	Embalmer, mortician, funeral director, manager
Retail stores	Pharmacist, pharmacy technician
Corrections facilities	Physician, nurse
State COVID-19 Shelter	Staff, transportation provider
Group homes for individuals in recovery in which COVID-19 cases are housed	Nurse, other medical staff
Group homes for individuals with high-risk comorbidities	Nurse, other medical staff

[1] This is not an exhaustive list of roles that qualify for Phase 1a vaccination in these settings. Job function will determine individuals who qualify as Phase 1a at-risk health workers.

In anticipation that the initial supply of vaccines will be severely limited, DPHS has further grouped at-risk health workers into three sub-categories of most, moderate and lowest risk. Only the most and moderate categories are included in Phase 1a:

i. Most risk –included in Phase 1a: Front line clinical staff who provide direct patient care and support staff with risk of exposure to bodily fluids or aerosols (e.g., ED, ICU, urgent care, respiratory therapists, occupational medicine, COVID-19 testing personnel, environmental services staff, security, etc.)

ii. Moderate risk –included in Phase 1a: Staff who have indirect or limited patient contact (e.g., PT/OT, food delivery personnel, clergy, on-unit unit clerks, COVID-19 entry screeners, in-person medical interpreters, patient registration, hospital parking valets, vaccinators, etc.)

iii. Lowest risk – included after Phase 1a: Administrative or other staff with no expected routine patient contact (e.g., medical records, hospital administration, billing, kitchen staff who do not deliver food, non-COVID-19 laboratory, etc.) These workers are critical to maintain health infrastructure. These workers will be vaccinated as early as possible once Phase 1a is completed.

Table 2 below shows categories of occupations within hospital or ambulatory care settings that potentially meet the definition of at-risk health worker, including examples of specific occupations and their risk level. The reason for this additional way to categorize at-risk health workers is to ease interface with typical Human Resources categorizations. A full list of occupations within each group in these settings is available in Appendix A. Risk stratification are included as a guide, but are dependent on context and work setting (e.g., physicians who exclusively telework should be considered at "lowest risk"). For further assistance making decisions for vaccinating at-risk health workers, consider Appendix B.

Table 2: Occupational categories, examples, and risk levels

Category	Example Occupations	Suggested Risk Level
**Clinical frontline workers**<br>*Those with direct patient contact*	Physician, nurse, physician's assistant, home care worker	Most
**Non-clinical frontline workers**<br>*Those with direct patient contact*	COVID-19 laboratorians, security guards, building cleaning workers	Most
**Other clinical**<br>*Those with some direct patient contact*	Occupational therapists, psychologists, diagnostic technicians, social workers	Moderate
**Food/Retail**<br>*Those with some direct patient contact*	Food delivery personnel, retail pharmacists	Moderate
**Food/Retail**<br>*Those without patient contact*	Cooks, cashiers, food preparation workers, dishwashers	Lowest
**Other non-clinical**<br>*Those without patient contact*	Maintenance workers, biological scientists, dispatchers	Lowest
**Administrative, clerical and financial services**<br>*Those without patient contact*	Human resources manager, billing clerk, budget analyst	Lowest
**Computing/IT**<br>*Those without patient contact*	Computer support specialists, database administrator, data entry worker	Lowest

II. Older Adults in Residential Care Settings

NH includes older adults in residential settings in Phase 1a due to the disproportionate impact of COVID-19 in adult residential care settings in NH. This population includes two subgroups:

1) Long term care facility (LTCF) and skilled nursing facility (SNF) residents
2) Assisted living facility (ALF) residents

Staff in these settings are considered at- risk health workers above and will also receive the vaccine in Phase 1a. Older adults living in independent living facilities, even those on the same grounds as a LTCF, will be vaccinated after 1a because their residence is non-congregate and have not incurred a disproportionate impact in NH.

III. First Responders

First responders are defined as follows:

- **Fire and emergency medical services (EMS):** All private, public, and volunteer fire and EMS personnel whose job function puts them at high risk of exposure to COVID-19
- **Law enforcement:** All certified or sworn law enforcement officers in NH, whether employed full- or part-time whose job function involve frequent public contact. This includes Federal Law Enforcement personnel working in NH who will not be vaccinated through federal programs. This does not include officers working in correctional facilities, which are included in a later phase. This also does not include security staff at nonclinical locations (e.g., retail facilities, academic institutions).

In anticipation that the initial supply of vaccines will be severely limited, DPHS has further grouped at-risk first responders into three sub-categories of most, moderate and lowest risk. Only the most and moderate categories are included in Phase 1a:

I. **Most risk** – first responders included in Phase 1a are those that are expected because of their professional role to have direct patient contact.
II. **Moderate risk** – first responders included in Phase 1a are those that are expected because of their professional role to have indirect patient contact with patients. These first responders provide support to the direct patient contact/care providers.
III. **Lowest risk** – included after Phase 1a: remainder of first responders. These workers are critical to maintain operational infrastructure. These workers will be vaccinated as early as possible once Phase 1a is completed.

NH COVID-19 responders: Individuals participating in pandemic response across a range of public and private sector entities who are at higher risk of acquisition of COVID-19 due their specific job function. This includes vaccinators, testing units, specimen collectors, public health personnel with contact to COVID-19 patients and their close contacts, and COVID-19 laboratory personnel.

Prioritization of Individuals within Phase 1a

First, administer vaccine to at-risk health workers and first responders who qualify as "most risk" before "moderate risk." Again, risk stratification is largely dependent on job function and work setting.

Then prioritize staff based on both occupational and personal risk. The categories shown below are not listed in priority order, but suggest considerations for prioritization within Phase 1a. These include but are not limited to staff:

- With high risk medical conditions (who choose to disclose)
- Over the age of 65
- Working on COVID-19 units
- Providing direct patient care
- Who have to work without adequate PPE
- Who are disproportionately affected by COVID-19 such as persons in racial and ethnic minority groups
- Those who have had confirmed COVID-19 in the previous 90 days.

APPENDIX A: Specific Occupations of Workers within Health Care Settings

This table is an expanded version of Table 2 above. This table includes categories of workers in health care settings and specific occupations within these categories. The purpose of this table is to aid vaccine planners in deciding who qualifies for vaccination and in what phase. Note this list does not include every occupation that may be present in a health care setting, but is provided as a guide.

Appendix A Table: Occupations by Category

Category and NH Risk Stratification	Occupations – Not in Alphabetical Order
**Clinical Frontline Workers –** Those with direct patient contact are at **most risk.** Those who work remote or do not have direct patient contact should not be included as most risk.	Athletic Trainers
	Dentists and other oral health practitioners
	Dental Hygienists and Assistants
	Direct Support Professionals
	Emergency Medical Responders, Technicians and Paramedics
	Health Practitioner Support Technologists and Technicians
	Licensed Practical and Licensed Vocational Nurses
	Genetic Counselors
	Medical Dosimetrists
	Hearing Aid Specialists
	Home Health Aides
	Hospice Aides
	Orthotists and Prosthetists
	Medical Assistants
	Medical Equipment Preparers
	Medical Transcriptionists
	Pharmacy Aides
	Phlebotomists
	Nurse Anesthetists
	Nurse Midwives
	Nurse Practitioners
	Nursing Assistants, Orderlies, and Psychiatric Aides
	Pharmacists
	Physician Assistants
	Physicians
	Registered Nurses
	Surgeons
	Therapists
**Non-clinical Frontline Workers -** Those with direct patient contact are at **most risk**	Clergy
	Counselors
	Personal Care Aides
	Community and Social Service Managers and Staff
	Passenger Vehicle Drivers
	Environmental service personnel
	Security Guards

**Other (clinical) –** Those who have some direct patient contact are at **moderate risk**	Acupuncturists
	Audiologists
	Chiropractors
	Clinical Laboratory Technologists and Technicians
	Dental and Ophthalmic Laboratory Technicians and Medical Appliance Technicians
	Diagnostic Related Technologists and Technicians
	Dietitians and Nutritionists
	Massage Therapists
	Occupational Health and Safety Specialists and Technician
	Occupational Therapists, Assistants, and Aides
	Opticians, Dispensing
	Optometrists
	Physical Therapists, Assistants, and Aides
	Podiatrists
	Psychologists
	Social Workers
	Barbers, Hairdressers, Hairstylists and Cosmetologists
**Food/Retail –** Those who have some direct patient contact are at **moderate risk**	Food Delivery Personnel
**Food/Retail -** Those who have no contact with patients are at **lowest risk**	Cashiers
	Cooks/Food preparation Managers and Staff
	Dishwashers
	Dining Room and Cafeteria Manager and Staff
	Food Service Managers
	Other Food Preparation and Serving Related Workers
**Other non-clinical -** Those who have no contact with patients are at **lowest risk**	Biological Scientists and Technicians
	Childcare Workers
	Couriers and Messengers
	Dispatchers
	Driver/Sales Workers and Truck Drivers
	Grounds Maintenance Workers
	Instructional Coordinators
	Laundry and Dry-Cleaning Workers
	Lawyers and other legal staff
	Librarians and Media Collections Specialists
	Mail Clerks and Mail Machine Operators
	Maintenance and Repair Workers, General
	Medical Scientists
	Parking Enforcement Workers
	Switchboard Operators, Including Answering Service

**Administrative, clerical and financial services - Those who have no contact with patients are at <u>lowest risk</u>**	Administrative Services and Facilities Managers and Staff
	Accountants, Bookkeeping, and Auditing Managers and Staff
	Buyers and Purchasing Managers and Staff
	Chief Executives
	Compensation and Benefits Managers and Staff
	Customer Service Representatives
	Eligibility Interviewers, Government Programs
	Financial Managers and Staff
	Office and Administrative Support Managers and Staff
	Fundraisers
	Human Resources Managers and Staff
	Marketing and Sales Managers and Staff
	Medical and Health Services Managers
	Medical Records Specialists, Health Technologists and Technicians
	Meeting, Convention, and Event Planners
	Property, Real Estate, and Community Association Managers
	Public Relations and Fundraising Managers and Staff
	Public Relations Specialists
	Receptionists and Information Clerks
	Secretaries and Administrative Assistants
	Shipping, Receiving, and Inventory Clerks
	Social and Community Service Managers
	Training and Development Managers and Staff
	Transportation, Storage, and Distribution Managers
**Computing/IT -** Those who have no contact with patients are at **<u>lowest risk</u>**	Computer, Information Systems and Database Managers and Staff
	Computer Support Specialists
	Data Entry and Information Processing Workers
	Health Information Technologists, Medical Registrars, Surgical Assistants, and Healthcare Practitioners and Technical workers, all others

APPENDIX B: Determining if Health Workers and First Responders are in Phase 1a

Algorithm to Determine if Workers in Health Care Settings* and First Responders are in Phase 1a

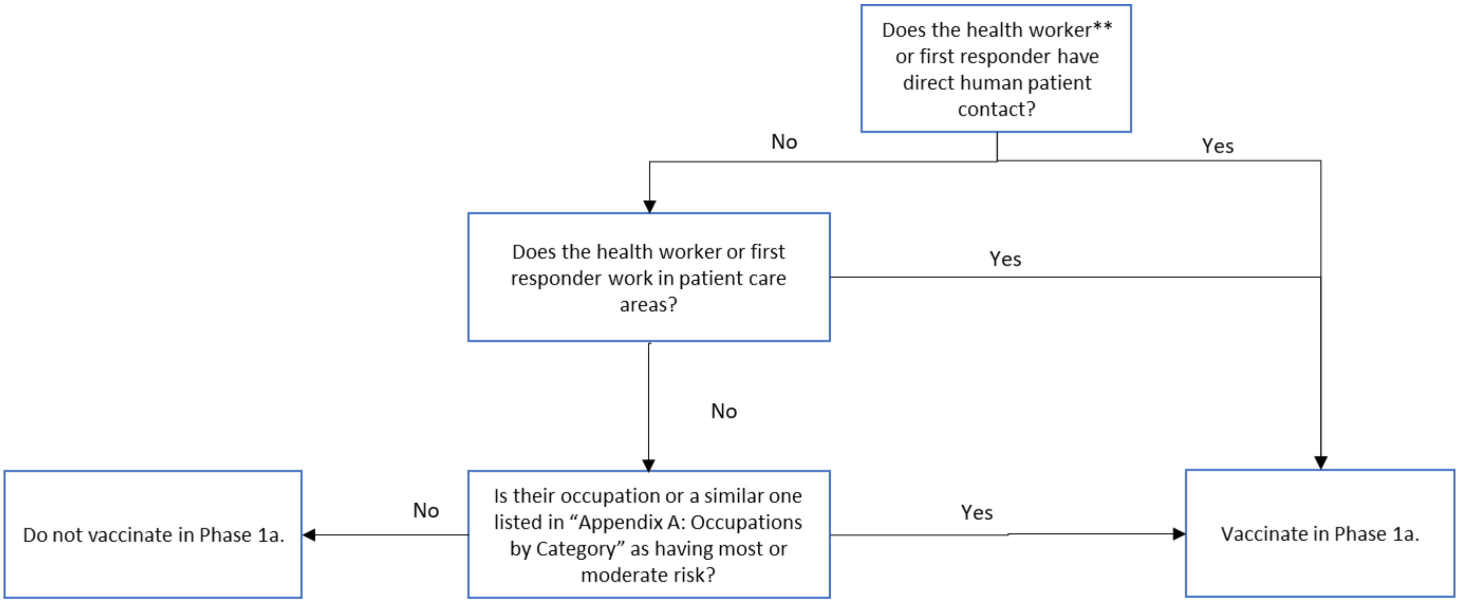

*Health care settings: These include but are not limited to acute and rehabilitation hospitals; ambulatory and urgent care clinics; dialysis centers; blood, organ, and tissue donation facilities; nursing homes; home care; school nurse offices and other settings (see Table 1).

**Health worker (and first responders with potential of patient contact as described above): Paid or unpaid workers in health care settings who care for patients (i.e., physician, nurse, etc.) or work in areas where patients are located (i.e., environmental services, on-unit clerks). This group will include traditional healthcare roles but also may include others who have received formal specialized medical training that enables them to do clinical care procedures. For example, school paraprofessionals are not routinely included within this definition of health workers unless they have received such specialized training. The person does not need to have earned a clinical degree, but are reasonably expected to have ongoing exposure to patients in the course of their work, including those who distribute or administer the COVID-19 vaccine such as pharmacists; plasma and blood donation workers; public health nurses; COVID-19 laboratorians; and morticians, funeral home workers and other death care professionals.
