# Supplementary material for: Ensuring Equitable COVID-19 Vaccine Allocation in New Hampshire: The First Eight Months toward a New Era
Source: Vaccines (Basel). 2022 Aug 29;10(9):1421. doi: 10.3390/vaccines10091421 (PMC9501825; doi:10.3390/vaccines10091421)
Supplement: Supplementary file 1 [file vaccines-10-01421-s001.zip › vaccines-1843494-supplementary/S5.pdf]

NH COVID-19 Vaccination Allocation Guidelines for Phase 1b

February 5, 2021

(changes in **red**)

NH is taking a phased approach to COVID-19 vaccine rollout, as described in the NH COVID-19 Vaccination Plan. The purpose of this document is to aid decision-making regarding Phase 1b populations. Guidance regarding Phase 1a was released December 8, and finalized phase descriptions can be found in the Vaccination Plan Summary. This document is dynamic, and subject to change, because vaccine efficacy and safety data continue to emerge and national guidance evolves (e.g., ACIP guidance). This document provides:

- An overview of the populations within phase 1b
- Principles to help make decisions about who should be included in phase 1b
- Guidance to further prioritize among these populations because there will be limited dose availability
- Description of the 10% equity allocation plan

Phase 1b: Population Overview

The New Hampshire Division of Public Health Services (DPHS) advises inclusion of the groups for vaccination under Phase 1b as shown in the graphic below. Beginning in Phase 1b, DPHS will also allocate 10% of available vaccine doses for disproportionately impacted populations.

People ≥ 65 years old	Medically vulnerable	Residents and staff of residential facilities for persons with intellectual and developmental disabilities	Corrections officers and staff working in correctional facilities	Health workers and first responders not already vaccinated
	Persons at significantly higher risk of morbidity or mortality, with 2 or more conditions (see list)<br><br>Family caregivers of those medically vulnerable persons, <16 years old (not eligible for vaccine)			

NH will also allocate 10% of vaccine for disproportionately impacted populations identified through the COVID-19 Community Vulnerability Index (CCVI)

I. People ≥ 65 Years Old: NH DPHS recommends vaccinating all adults 65 years and older, because of significantly increased risk of severe COVID-19 and death compared to those who are younger.

When vaccine supply is limited and circumstances allow, DPHS recommends (but does not require) that administration can be prioritized among older adults within phase 1b, such as:

Vaccinate those who are either:	With more medical comorbidities before those with fewer
	75 years of age and older before persons aged 65-74 years of age
	Living in residential/congregate settings before those who are living in a private home
	Have suffered significant mental health and/or physical decline because of "Safer at Home" guidelines because of a lack of socialization before those who have not.[1]
	Without capacity to limit contact with outside persons before those with such capacity
	Without confirmed COVID-19 within the previous 90 days before those with confirmed COVID-19 in the previous 90 days

II. Medically Vulnerable: NH DPHS recommends vaccinating persons with comorbid and underlying conditions that put them at *significantly* higher risk as defined by having two or more of the specified underlying medical conditions below that increase the risk of severe COVID-19 or death. Please note, this is not an exhaustive list of medical conditions, and providers should use clinical discretion identifying individual patients who are at increased risk of morbidity or mortality from COVID-19 based on additional guidance below.

List of Underlying Medical Conditions (modified from CDC):
Phase 1b: Two or more conditions

- Cancer
- Chronic Kidney Disease
- COPD (Chronic Obstructive Pulmonary Disease) and other high-risk pulmonary disease
- Down Syndrome
- Heart Conditions, such as heart failure, coronary artery disease, or cardiomyopathies
- Immunocompromised states
- Obesity (body mass index of 30 kg/m or higher)
- Pregnancy
- Sickle cell disease
- Type 2 Diabetes Mellitus

Note: DPHS allows a health care provider to vaccinate any patient assessed to have significant risk for severe illness due to co-morbidities, even if not listed here. This list does not include every condition that might increase one's risk for developing severe illness from COVID-19, such as those for which evidence may be limited (e.g., rare conditions or combinations of conditions).

[1] Together, COVID-19 vaccination and following recommendations for how to protect yourself and others will offer the best protection from getting and spreading COVID-19. It will be important for everyone to continue using all the tools available to help stop this pandemic, like wearing a mask, washing hands often, and staying at least 6 feet away from others (CDC).

Practical suggestions (not requirements) regarding identifying patients who are at increased risk of severe COVID-19 and death based on the listed conditions intended for Phase 1b prioritization:

- **Cancer**: Patients who have current cancer or who were recently treated for cancer which continues to cause increased susceptibility to severe COVID-19. This category is not intended to include people with a history of cured cancer, even if at risk of recurrence, or people with a family history or genetic testing suggesting increased risk for cancer. Additionally, vaccination for persons with organ or blood cancers should be prioritized over those with superficial non-metastasized skin cancer pending local treatments.
- **Chronic kidney disease**: Patients who have chronic sustained elevated creatinine indicative of renal dysfunction, with associated health consequences such as fluid overload and blood electrolyte abnormalities (e.g., low calcium levels, high potassium levels, and high phosphorus levels), or have been diagnosed end-stage renal disease, those on hemo- or peritoneal-dialysis. We suggest delaying vaccination for patients who have had acute kidney disease due to a reversable condition (e.g., pre-renal azotemia).
- **Chronic obstructive lung disease (COPD) and other high risk pulmonary disease**: Patients who have any of the group of diseases that comprise COPD, including emphysema and chronic bronchitis, or who have other chronic lung diseases that are actively compromising pulmonary function, such as chronic bronchiectasis, idiopathic pulmonary fibrosis, cystic fibrosis, lung transplantation, and moderate/severe asthma that requires daily medication for control. Assuming no other qualifying comorbidities, we suggest delaying vaccination for those with less severe pulmonary diseases such as a remote history of asthma, smoking with no known pulmonary complications, history of treated bacterial or viral pneumonia, and/or those who have a pulmonary disease but normal lung function tests.
- **Down syndrome**: Patients who have trisomy 21, regardless of degree of disability. There is limited data regarding other chromosomal abnormalities (e.g., Edward's syndrome [trisomy 18], or Cri du chat syndrome [5p minus syndrome]), anoxic birth injury (e.g., cerebral palsy), birth defects (e.g., spina bifida) or severe autism spectrum disorder (ASD) so DPHS suggests including such patients with inability to independently perform activities of daily living or require mechanical support (e.g., mechanical ventilation, feeding tubes). Note those with ASD is a large and diverse group of patients, most of whom are not thought to be at increased risk from COVID-19.
- **Heart conditions and other cardiovascular and cerebrovascular disease**: Patients who have been diagnosed with heart failure (either reduced ejection fraction or heart failure with preserved ejection fraction), coronary artery disease (e.g., myocardial infection, stable or unstable angina, aortic aneurysm and dissection), cardiomyopathies, arrhythmias, pulmonary hypertension, repaired or symptomatic congenital heart defects, valvular disease, other atherosclerotic conditions (e.g., peripheral vascular/arterial disease), valve disease (e.g., rheumatic heart disease, significant stenoses and regurgitation). Also include those with cerebrovascular disease, including people with stokes who have impaired ability to carry out activities of daily living (ADLs). Delay those with family history of such diseases, recovered infective endocarditis, and uncomplicated mitral valve prolapse.
- **Immunocompromised states**: Patients who are immunocompromised due to solid organ or bone marrow transplant; primary immunodeficiency (e.g., severe combined immunodeficiency, idiopathic CD4 lymphopenia); HIV or AIDS, especially people with CD4 cell count less than 200 cells/mm3 or not on antiretroviral treatment; prolonged use of corticosteroids; or people who are undergoing a work-up for planned organ or bone marrow transplantation can be prioritized due to anticipated immunosuppression. To date, there is not evidence that patients with autoimmune disease are at increased risk of worse outcomes even if taking disease-modifying antirheumatic drugs (DMARDS, such as TNF-alpha inhibitors or

JAK inhibitors). In fact, some of these drugs, like hydroxychloroquine, dexamethasone, and tocilizumab have been studied for treatment of COVID-19. .

- **Obesity:** current BMI >30 kg/m
- **Pregnancy**: Women in any trimester of pregnancy. Delay those who are post-partum (whether or not lactating) or intending pregnancy.
- **Sickle cell disease**: Patients who have hemoglobin disorders including sickle cell disease and thalassemia.
- **Diabetes**: Patients who have type 2 diabetes, requiring oral hypoglycemic medication or regular insulin. The data for increased risk is less robust for those with type 1 diabetes, but clinicians should use clinical judgement and can include patients with type 1 diabetes in Phase 1b vaccination, because of frequent comorbidities and significant health risks posed by both type 1 and type 2 diabetes. We suggest delaying vaccination for those with pre-diabetes, and those with a history of gestational diabetes who have returned to euglycemia post-partum.

Currently, no available vaccine formulations are approved for use in persons under 16 years of age. Therefore, the family caregivers who provide regular direct care to children under 16 years old whose provider has attested that the child has two more conditions that put them at significantly higher risk for severe COVID-19 disease or death may be vaccinated in Phase 1b to offer protection to the child. Family caregivers of these medically vulnerable children under 16 years of age may be eligible for vaccine if:

1. The medically vulnerable child is primarily isolated at home, where the caregivers would be the most likely source of exposure to COVID-19. The reasons for being homebound* include:
    a. The person's doctor believes that their health or illness could get worse if they leave the home;
    b. The person requires the help of another person and/or medical equipment to leave the home, or find it difficult to leave the home and typically cannot do so; and
2. These family caregivers are the primary provider of the services to their children under the age of 16; and
3. The children are authorized for private duty nursing (PDN) and other home health care supports within the home that meet an institutional level of care (LOC) due to the child's chronic health conditions.

*Note being homebound includes going out for medical appointments or treatment or for short periods of time or for special non-medical events.

In order to be scheduled for vaccination, these family caregivers must have their child's healthcare provider submit a medical verification form to NH DHHS or enter into VAMS (for those providers with access to VAMS).

When vaccine supply is limited and circumstances allow, DPHS recommends that administration can be prioritized among the medically vulnerable within phase 1b, such as:

Vaccinate those who are either:	65 years or older before those who are younger
	With more medical comorbidities before those with fewer
	Living in residential/congregate settings before those who are living in a private home
	Without capacity to limit contact with outside persons before those with such capacity
	Without confirmed COVID-19 within the previous 90 days before those with confirmed COVID-19 in the previous 90 days

III. Residents and staff of residential facilities for persons with intellectual and developmental disabilities

Paid or unpaid staff working and people living in residential facilities for persons with intellectual and developmental disabilities should also be prioritized for vaccination in Phase 1b due to their increased risk of transmitting infection to patients at higher risk of mortality and severe morbidity, much like staff in long-term care facilities.

When vaccine supply is limited and circumstances allow, DPHS recommends that administration can be prioritized among these individuals within phase 1b, such as:

Vaccinate those who are either:	65 years or older before those who are younger
	With more medical comorbidities before those with fewer
	Without capacity to limit contact with outside persons before those with such capacity
	Without confirmed COVID-19 within the previous 90 days before those with confirmed COVID-19 in the previous 90 days

IV. Corrections officers and staff working in correctional facilities

Correctional facility officers and all other staff (paid or not paid) should be vaccinated in Phase 1b, due to 1) their work in situations where the risk of SARS-CoV-2 transmission is higher, 2) being at an elevated risk of transmitting infection within the facility, and 3) their functioning much like a first responder within this setting.

When vaccine supply is limited and circumstances allow, DPHS recommends that administration can be prioritized among these individuals within phase 1b, such as:

Vaccinate those who are either:	65 years or older before those who are younger
	With more medical comorbidities before those with fewer
	Have more direct contact with residents than those without contact
	Full- before part-time
	Without confirmed COVID-19 within the previous 90 days before those with confirmed COVID-19 in the previous 90 days

V. Health workers and first responders:

As a continuation of Phase 1a prioritization, health workers and first responders not vaccinated in Phase 1a, should be vaccinated during Phase 1b. See Phase 1a guidance for definitions of the workers who remain eligible to be vaccinated in Phase 1b.

When vaccine supply is limited and circumstances allow, DPHS recommends that administration can be prioritized among these individuals within phase 1b, such as:

Vaccinate those who are either:	65 years or older before those who are younger
	With more medical comorbidities before those with fewer
	Have more public contact than those with less contact
	Without confirmed COVID-19 within the previous 90 days before those with confirmed COVID-19 in the previous 90 days

Phase 1b: Equity Allocation

In accordance with the National Academies of Sciences, Engineering, and Medicine (NASEM) recommendations, beginning in Phase 1b, NH will allocate 10% of the available state vaccine supply to geographic areas that are highly vulnerable to COVID-19. DPHS will:

- Identify communities through the COVID-19 Community Vulnerability Index (CCVI) and US Census data
- Distribute vaccines predominately through mobile vaccination clinic sites
- Coordinate this distribution through the NH Regional Public Health Networks at sites that are (1) familiar and accessible to the target population and (2) geographically positioned for easy access on foot or through public transportation when available.
- Initially provide vaccine to NH's racial and/or ethnic minority community then include other vulnerable populations, such as those that are geographically isolated or those living in economic hardship.
- Reserve vaccine for use in targeted response in these identified census tract areas if needed.

When vaccine supply is limited and circumstances allow, DPHS recommends that administration can be prioritized among populations defined as disproportionally impacted racial and ethnic minority communities within phase 1b, such as:

Vaccinate those who either:	Are 65 years or older before those who are younger
	Have more medical comorbidities before those with fewer
	Live in multi-generational households before those who do not
	Have reduced opportunities for accessing healthcare, transportation or other key supportive services necessary to access vaccination
	Have more public contact than those with less contact
	Without confirmed COVID-19 within the previous 90 days before those with confirmed COVID-19 in the previous 90 days
	Have no opportunity to receive vaccine through other locations or providers before those who do
