# Supplementary material for: Ensuring Equitable COVID-19 Vaccine Allocation in New Hampshire: The First Eight Months toward a New Era
Source: Vaccines (Basel). 2022 Aug 29;10(9):1421. doi: 10.3390/vaccines10091421 (PMC9501825; doi:10.3390/vaccines10091421)
Supplement: Supplementary file 1 [file vaccines-10-01421-s001.zip › vaccines-1843494-supplementary/S6.pdf]

NH COVID-19 Vaccination Allocation Guidelines for Phase 2
March 3, 2021

New Hampshire (NH) is taking a phased approach to COVID-19 vaccine rollout, as described in the NH COVID-19 Vaccination Plan. The purpose of this document is to aid decision-making regarding Phase 2 populations. Finalized phase descriptions can be found in the Vaccination Plan Summary. This document provides:

- An overview of the populations within phase 2
- Principles to help make decisions about who should be included in phase 2
- Guidance to further prioritize among these populations because there will be limited dose availability

Phase 2: Population Overview

The NH Division of Public Health Services (DPHS) advises inclusion of the groups for vaccination under Phase 2 as shown in the graphic below. As has already begun in Phase 1b, DPHS will allocate 10% of available vaccine doses for disproportionately impacted populations.

Phase 2a

- All staff working in New Hampshire:
  - Schools serving children in kindergarten through grade 12;
  - Child care and Head Start and Early Head Start programs; and
  - Youth recreation camps

Phase 2b

- New Hampshire residents 50 to 64 years of age

NH will allocate 10% of vaccine for disproportionately impacted populations identified through the COVID-19 Community Vulnerability Index (CCVI)

Detailed Description of School and Child Care and Youth Camp Populations Within Phase 2a

The below groups within Phase 2a represent a diverse population, and DPHS suggests that administrators within the described institutions enable their staff to equally access vaccination. For example, consider facility-wide language-appropriate campaigns to raise awareness about staff eligibility, and provide support for those without access to a computer or reliable internet to register, if needed.

Given that child care and youth camp programs often employ teenagers, it is important to note that the vaccination eligibility criteria outlined below is also dependent on availability of vaccines approved for use in the staff person's age group. While currently there are only vaccines approved for use in persons 16 years of age and older, we expect that vaccines will eventually be approved for younger age groups. Individuals in Phase 2a too young to be vaccinated now should receive the vaccine as soon as a vaccine is approved and becomes available for use in their age group.

I. K-12 School Staff: K-12 schools include public district, charter, private, and other non-public schools. Consistent with the National Academies of Sciences, Engineering, and Medicine, NH defines school staff as workers receiving compensation as classroom teachers, librarians, administrators, office staff, para educators, custodial staff, other education support professionals, food services personnel, bus drivers and bus monitors. School nurses are also school staff, but were eligible for vaccination in phase 1a. Staff considered appropriate for vaccination may be full or part time (including substitute teachers). Student teachers are also eligible if they are at the school to meet requirements of their academic program.

II. Child Care Program Staff: Child care program staff include workers receiving compensation from:

- A licensed child care setting;
- A license-exempt setting enrolled with the NH DHHS - Bureau of Child Development;
- A municipal recreation program, including after-school and summer recreation programs; or
- A recreational program providing before and/or after school, vacation, or summer youth programming for children 6 years of age or older offered by a school or religious group, the Boys and Girls Clubs of America, Girls, Incorporated, the YMCA, or the YWCA, provided that the program does not operate in a private home.

Child care program staff includes office, kitchen and janitorial staff; contract employees or licensed self-employed individuals; whose activities involve the care or supervision of children for a child care program. Child care program personnel as defined in the NH Child Care Licensing Rules include:

• Assistant group leader	• Family child care worker	• Office staff
• Assistant teacher	• Group Leader	• Site coordinator
• Associate teacher	• Project leader	• Site director
• Custodial staff	• Household member	• Substitute
• Family child care assistant	• Junior helper*	
Family child care provider	• Lead teacher	

Student child care providers are also eligible if they are reporting to the child care program to meet requirements of their academic program.
*Subject to availability of vaccine approved for use in persons younger than age 16.

III. <u>Head Start and Early Head Start Staff</u>: Head Start and Early Head Start programs promote the school readiness of infants, toddlers, and preschool-aged children from low-income families. Services are provided in a variety of settings including centers, family child care, and children's own home. Staff include workers receiving compensation in roles as described in Head Start Staff Qualifications:

• Head Start/Early Head Start directors; program directors and assistant directors<br>• Head Start managers:<br>○ Education/child development<br>○ Health services/nutrition<br>○ Disability services<br>○ Family services<br>• Office staff<br>• Child development specialists	• Head Start Center-based preschool teachers and assistant teachers<br>• Early Head Start Center-based infant and toddler teachers<br>• Family services staff/family advocates<br>• Home visitors<br>• Head Start/Early Head Start program coaches	• Health, oral health, early childhood mental health and nutrition staff<br>• Food services staff (e.g., cooks)<br>• Head Start/Early Head Start center custodial staff<br>• Head Start/Early Head Start consultants and specialists (e.g., physical therapist, occupational therapist, speech and language therapist, social work, etc.)

Student child care providers are also eligible if they are reporting to the child care program to meet requirements of their academic program.

IV. Youth Recreation Camp Staff

Youth recreation camp staff include workers currently receiving or hired to receive compensation in 2021 in roles as described in the Youth Recreation Camps administrative rule:

• Director<br>• Counselor<br>• Counselor in-training*<br>• Junior counselor*	• Activity leader<br>• Instructor	• Health care staff<br>• Kitchen staff<br>• Maintenance staff<br>• Special recreation staff

*Subject to availability of vaccine approved for use in persons younger than age 16.

Detailed Description of 50-64 year old Populations Within Phase 2b

People 50-64 years of age: NH DPHS recommends vaccinating all adults ages 50-64, because of an increased risk of severe COVID-19 and death compared to those who are younger. When vaccine supply is limited and circumstances allow, DPHS recommends (but does not require) that administration can be prioritized among older adults within phase 2b, such as:

Vaccinate those who are:	Older before those who are younger
	With medical comorbidities* before those with none
	Living in residential/congregate settings that had not previously qualified for vaccination before those who are living in a private home
	Living in multi-generational homes before those who do not
	Have suffered significant mental health and/or physical decline because of "Safer at Home" guidelines because of a lack of socialization before those who have not.1[1]
	Without capacity to limit contact with outside persons before those with such capacity
	Without confirmed COVID-19 within the previous 90 days before those with confirmed COVID-19 in the previous 90 days

*Medical comorbidities have been described in the Phase 1b Technical Assistance, and include:

- Cancer
- Chronic Kidney Disease
- COPD (Chronic Obstructive Pulmonary Disease) and other high-risk pulmonary disease
- Down Syndrome
- Heart Conditions, such as heart failure, coronary artery disease, or cardiomyopathies
- Immunocompromised states
- Obesity (body mass index of 30 kg/m or higher)
- Pregnancy
- Sickle cell disease
- Type 2 Diabetes Mellitus

[1] Together, COVID-19 vaccination and following recommendations for how to protect yourself and others will offer the best protection from getting and spreading COVID-19. It will be important for everyone to continue using all the tools available to help stop this pandemic, like wearing a mask, washing hands often, and staying at least 6 feet away from others (CDC).
